# Mass–abundance scaling in avian communities is maintained after tropical selective logging

**DOI:** 10.1002/ece3.6066

**Published:** 2020-02-29

**Authors:** Cindy C. P. Cosset, James J. Gilroy, Umesh Srinivasan, Matthew G. Hethcoat, David P. Edwards

**Affiliations:** ^1^ Department of Animal and Plant Sciences University of Sheffield Sheffield UK; ^2^ School of Environmental Sciences University of East Anglia Norwich UK; ^3^ Program in Science, Technology and Environmental Policy Woodrow Wilson School for Public and International Affairs Princeton University Princeton NJ USA; ^4^ School of Mathematics and Statistics University of Sheffield Sheffield UK; ^5^ Grantham Centre for Sustainable Futures University of Sheffield Sheffield UK

**Keywords:** biodiversity, birds, local size–density relationship, mass–abundance relationship, production forest, size distribution

## Abstract

1. Selective logging dominates forested landscapes across the tropics. Despite the structural damage incurred, selectively logged forests typically retain more biodiversity than other forest disturbances. Most logging impact studies consider conventional metrics, like species richness, but these can conceal subtle biodiversity impacts. The mass–abundance relationship is an integral feature of ecological communities, describing the negative relationship between body mass and population abundance, where, in a system without anthropogenic influence, larger species are less abundant due to higher energy requirements. Changes in this relationship can indicate community structure and function changes.
2. We investigated the impacts of selective logging on the mass–abundance scaling of avian communities by conducting a meta‐analysis to examine its pantropical trend. We divide our analysis between studies using mist netting, sampling the understory avian community, and point counts, sampling the entire community.
3. Across 19 mist‐netting studies, we found no consistent effects of selective logging on mass–abundance scaling relative to primary forests, except for the *omnivore* guild where there were fewer larger‐bodied species after logging. In eleven point‐count studies, we found a more negative relationship in the whole community after logging, likely driven by the *frugivore* guild, showing a similar pattern.
4. Limited effects of logging on mass–abundance scaling may suggest high species turnover in logged communities, with like‐for‐like replacement of lost species with similar‐sized species. The increased negative mass–abundance relationship found in some logged communities could result from resource depletion, density compensation, or increased hunting; potentially indicating downstream impacts on ecosystem functions.
5. *Synthesis and applications*. Our results suggest that size distributions of avian communities in logged forests are relatively robust to disturbance, potentially maintaining ecosystem processes in these forests, thus underscoring the high conservation value of logged tropical forests, indicating an urgent need to focus on their protection from further degradation and deforestation.

## Graphical abstract

Most studies of logging impacts consider conventional metrics such as species richness, which can conceal subtle impacts on biodiversity such as the mass–abundance relationship, where changes in this relationship can indicate shifts in community structure and function. We investigated the impacts of selective logging on the mass–abundance scaling of avian communities by conducting a meta‐analysis to examine its pantropical trend, dividing the analysis between mist‐netting and point‐count studies. We largely found no consistent effects of selective logging in the mist‐netting studies, except for the omnivore guild where the mass–abundance relationship became more negative after logging; a pattern also found in the whole point‐count community, likely driven by the frugivore guild. Our results suggest that avian size distributions are relatively robust to logging, potentially maintaining ecosystem processes in these forests, thus underscoring the high conservation value of logged forests, indicating an urgent need to focus on their protection from further degradation and deforestation.

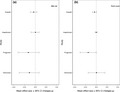

## 1 INTRODUCTION

Selective logging is the dominant anthropogenic activity in the tropics (Edwards, Tobias, Sheil, Meijaard, & Laurance, 2014), responsible for degrading over 390 million hectares of tropical forests globally (Asner, Rudel, Aide, Defries, & Emerson, 2009; Blaser, Sarre, Poore, & Johnson, 2011), with extensive additional illegal logging (Lawson & MacFaul, 2010). Despite the structural damage to forests caused by logging (Hawthorne, Sheil, Agyeman, Abu Juam, & Marshall, 2012; Osazuwa‐Peters, Chapman, & Zanne, 2015; Putz, Sist, Fredericksen, & Dykstra, 2008), selectively logged forests retain more biodiversity compared with other forest disturbances (Edwards, Magrach, et al., 2014; Gibson et al., 2011; Putz et al., 2012), although community composition is altered compared with primary forests and impacts on biodiversity are more adverse at higher logging intensities (Burivalova, Sekercioglu, & Koh, 2014; Edwards et al., 2011; Edwards, Magrach, et al., 2014; Martin, Newton, Pfeifer, Khoo, & Bullock, 2015). However, studies using conventional metrics (i.e., species richness and community composition; Burivalova et al., 2014; Costantini, Edwards, & Simons, 2016) can conceal hidden impacts on ecosystem functioning. For instance, in Borneo, high‐intensity logging (twice‐logged) resulted in a half‐trophic level increase in the trophic position of nine of ten understory bird species (Edwards et al., 2013), indicating that these species were feeding from higher up the food chain, via a switch to a more invertebrate‐rich diet. There is thus a need to investigate the impacts of selective logging on ecosystem properties that represent community function to better understand the future of biodiversity in selectively logged forests.

Mass–abundance scaling describes the negative relationship between a species' body mass and population abundance (Damuth, 1981), where, in a system without anthropogenic pressures, larger species typically occur at lower abundances due to their higher energy and resource requirements compared with smaller species. Because body mass determines metabolic rate, and thus resource use, the mass–abundance relationship describes resource partitioning within an ecosystem (White, Ernest, Kerkhoff, & Enquist, 2007) and underpins food‐web stability in ecological systems (O'Gorman & Emmerson, 2011; Otto, Rall, & Brose, 2007; Riede et al., 2011). Shifts in the mass–abundance relationship after land‐use changes can indicate alterations in the structure and function of ecological communities. For example, selective logging can impose primary and secondary impacts on ecosystems. Its primary impacts are that larger species tend to be more vulnerable to selective logging (Burivalova et al., 2015), which could lead to losses in large‐seed dispersers. The secondary impacts from these losses include lower recruitment in large‐seeded plants (Culot, Bello, Batista, do Couto, & Galetti, 2017), which greatly impede forest regeneration (Gardner, Bicknell, Baldwin‐Cantello, Struebig, & Davies, 2019; Osazuwa‐Peters et al., 2015) and carbon stocking (Bello et al., 2015; Osuri et al., 2016; Peres, Emilio, Schietti, Desmouliere, & Levi, 2016).

To our knowledge, only two studies have directly evaluated the impacts of selective logging on the mass–abundance relationship of ecological communities, both on avian communities (Sreekar et al., 2015; Srinivasan, 2013), and these showed contrasting results. Srinivasan (2013) found that the mass–abundance relationship of understory insectivorous bird communities (body size ranging 6.1–71.3 g) became more negative (i.e., fewer larger‐bodied species) as logging intensity increased in the Himalaya. This suggests that density compensation occurs when resource declines cause larger species to become rarer, allowing smaller species to access resources previously monopolized by larger species and increase in abundance (MacArthur, Diamond, & Karr, 1972). In contrast, Sreekar et al. (2015) found no changes in the mass–abundance relationship between primary forests, degraded forests, and agricultural lands in Sri Lanka, which was likely due to the high species turnover observed in each land‐use type. These contrasting results invoke the need for a meta‐analysis, where data from appropriate studies across the tropics will be used to determine the global trend of this relationship.

We investigated the impacts of selective logging on the mass–abundance scaling of avian communities—which are well‐known taxonomically (Jetz, Thomas, Joy, Hartmann, & Mooers, 2012), good indicators of responses to environmental change in other taxa (Edwards, Magrach, et al., 2014) and important for ecosystem functioning (Sekercioglu, 2006)—by conducting a meta‐analysis to examine the overall pantropical trend of this relationship. We use the local size–density relationship (LSDR) between the average body mass of a species and the abundance of the species, with all abundances coming from localized study areas. We measured the slope of the upper bound of this mass–abundance relationship (Srinivasan, 2013) since the upper bound represents the maximum potential abundance of a species of a certain body size, typically between the 75th percentile and the 95th percentile of the mass–abundance relationship. This upper bound is measured because (a) the LSDR is determined by processes that influence resource allocation between species (White et al., 2007) and, therefore, the upper bound is likely to be energetically limiting (Blackburn, Lawton, & Perry, 1992); and (b) local assemblages tend to contain species with lower population sizes compared with larger global‐scale communities (Brown, Mehlman, & Stevens, 1995) as they contain only a subset of the global population size. We tested the hypothesis that logging typically decreased the upper‐bound slope of the mass–abundance relationship, relative to primary forest, due to disproportionate effects on the abundance of large‐bodied species (Srinivasan, 2013). We also investigated the impacts of selective logging on mass–abundance scaling within different avian foraging guilds, given that guilds differ in their rates of energy consumption as well as energy availability (Russo, Robinson, & Terborgh, 2003) and that foraging guilds often respond differently to land‐use change (Sreekar et al., 2015).

## 2 MATERIALS & METHODS

### 2.1 Data collection

**Figure 1:**
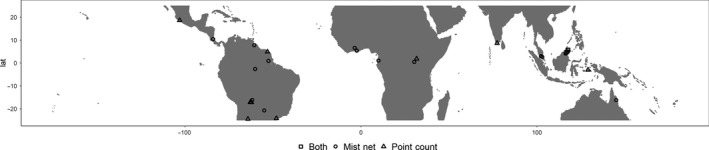
Distribution of the nineteen mist‐net studies and eleven point‐count studies across the tropics

Data were obtained from 30 studies (19 studies using mist‐netting methods and 11 studies using point‐count methods) that contained information on abundance or capture rate for avian species in both selectively logged forests and old‐growth primary forest controls across the tropics (Figure 1, Table S1 and S2). The online Web of Science database was used to search for studies with the keywords [“selective logging” OR forestry OR “secondary forest” OR “regenerating forest”] AND [bird* OR avian OR aves] AND [mass OR abundance OR number OR “capture rate” OR density]. This search was refined by [tropic*] and [“mist‐net” OR “point‐count”] resulting in 80,156 studies. We further refined the search to only include studies with topics such as environmental sciences, ecology, forestry, zoology, and biodiversity conservation, leaving us with 525 studies. We then supplemented the search using two more Google Scholar searches with the keywords; search 1: “selective logging”, bird*, avian, aves, mass, abundance, number, “capture rate”, density, “mist‐net*”, “point‐count*”, tropic*; search 2: “regenerating forest”, bird*, avian, aves, mass, abundance, number, “capture rate”, density, “mist‐net*”, “point‐count*”, tropic*. Search 1 resulted in 774 studies and search 2 returned 215 studies. This left us with a total of 1,514 studies, and after removing duplicates, we were left with 1,395 studies. Excluding studies based on title reduced the collection to 676 studies, and excluding the remaining studies based on abstract resulted in 211 studies. All searches were conducted between 4 April 2019 and 18 April 2019.

Of these 211 studies, studies were only included during full‐text screening if they were (a) conducted in the tropics (between 23.43706°N and 23.43706°S), (b) conducted in closed‐canopy forests, (c) used mist netting or point counts to sample birds, (d) presented species‐specific abundance estimates in both selectively logged forests and old‐growth primary forests, and (e) mist‐net and point‐count datasets (if both included) could be separated. This resulted in a total of 47 studies, for one of which the author no longer had the abundance dataset, another one for which the sole author (Johns, 1996) was uncontactable, and for 15 of which we did not get a response from the authors we contacted.

This left us with 30 studies (19 mist‐netting studies and 11 point‐count studies; see Table S1 and S2 for information on each study). Where available, mass was obtained from individual studies, and for studies in which no information on mass was provided (or where masses were missing for some species), we used Dunning's CRC Handbook of Avian Body Masses (2008) and Handbook of the Birds of the World Alive (del Hoyo, Elliott, Sargatal, Christie, & Juana, 2017). The data from two of these studies (Hawes, Barlow, Gardner, & Peres, 2008; Wunderle, Henriques, & Willig, 2006) were split and analyzed separately as they contained data from different habitats, where each habitat type contained a distinct avian community. This resulted in 21 separate mist‐netting studies.

### 2.2 Quantile regression

The mist‐netting data and point‐count data were analyzed separately. To study the impacts of selective logging on the mass–abundance scaling of avian communities across the tropics, a meta‐analysis was conducted on differences in the slope of the upper bound of mass–abundance relationships between logged forests and primary forest controls. The abundance or capture rate for each species was standardized within each study and within each habitat type (primary forest or logged forest) to obtain the relative standardized abundance:

$$\text{Relative}\;\text{Standardized}\;\text{Abundance}=\frac{x-\bar{x}}{\sigma }$$

*x* = Abundance

$\bar{x}$ = Mean abundance

σ = Standard deviation of abundance

Using relative standardized abundance rather than abundance or capture rate will affect the intercept but not the slope of the mass–abundance relationship. For each study and habitat type (primary forest or logged forest), the slopes of the upper bound of the mass–abundance relationship was estimated using quantile regressions in the R software (R Core Team, 2019) package quantreg (Koenker, 2017). Quantile regression enables the quantification of information from the boundaries of polygonal relationships (Scharf, Juanes, & Sutherland, 1998) and can identify factors that limit species' responses (Vaz et al., 2008). Standardized relative abundance was log10 (*y* + 1) transformed in the mist‐net data and log10 (*y* + 2) transformed in the point‐count data, while species mass was log10 transformed to obtain a straight line upper bound on the mass‐abundance relationship. The upper bound of the polygonal mass–abundance relationship is likely to represent an energetic limit on abundance (Blackburn & Gaston, 1997).

### 2.3 Meta‐analysis

For each paired logged and primary slope estimate, the mean difference effect size, Hedges' *g*, was calculated using the compute.es package (Del Re, 2013). Studies were weighted by the inverse of their variance so that smaller studies or those with high uncertainty contribute less to estimated effects. The average effect size was then calculated using the random‐effects model in the MAd package (Del Re & Hoyt, 2014). To test for the effect of elevation and geographic region (continent) on the effect sizes, a meta‐regression was performed with elevation and continent using the MAd package (Del Re & Hoyt, 2014). The extent of heterogeneity was tested using the *I* ^2^ statistic.

Publication bias was tested for using two methods in the metafor package (Viechtbauer, 2010). First, publication bias was examined visually using a funnel plot of effect size (Figure S9, Figure S10) and the second method was using Rosenthal's Fail‐Safe N analysis. This meta‐analysis was repeated using slope estimates from a range of regression quantiles (0.75, 0.8, 0.85, 0.9, 0.95). All analyses were done using the R software (R Core Team, 2019).

### 2.4 Guild analyses

To examine the impacts of selective logging on the mass–abundance scaling of different species foraging guilds, each species was first assigned to a foraging guild (insectivore, frugivore, omnivore, carnivore, and granivore; see Table S3 for more information) based on the categorization used in the EltonTraits 1.0 database (Wilman et al., 2014). This resulted in only three foraging guilds, *insectivore*, *frugivore*, and *omnivore*, containing enough data after removing studies where there were less than ten species per study and per habitat type (i.e., less than ten species in either primary forest or logged forest). The above meta‐analysis methods were then conducted separately for *insectivore*, *frugivore*, and *omnivore* foraging guilds. The resulting studies for each guild are as follows: *insectivore*: 19 mist‐netting studies and eleven point‐count studies; *frugivore*: ten mist‐netting studies and seven point‐count studies; and *omnivore*: nine mist‐netting studies and seven point‐count studies.

## 3 RESULTS

There was a small degree of within‐study heterogeneity (*I* ^2^: 0.004%–33.43%) in the 0.75 quantile models (mist net: *frugivore*; point count: *Overall*, *frugivore*), 0.8 quantile models (point count: *frugivore*, *omnivore*), 0.85 quantile models (point count: *frugivore*, *omnivore*), 0.9 quantile models (mist net: *frugivore*, *omnivore*; point count: *Overall*, *frugivore*, *omnivore*), and 0.95 quantile models (mist net: *Overall*, *frugivore*, *omnivore*; point count: *Overall*, *insectivore*, *frugivore*, *omnivore*). However, there was a large degree of uncertainty in these I^2^ estimates, which is to be expected due to the small amount of studies. Publication bias was detected for the 0.95 quantile *Overall* model (mist net: *p* = .044, Fail‐safe *N* = 2, Figure S9; point count: *p* = .01, Fail‐safe *N* = 11, Figure S10), the 0.9 *omnivore* model (mist net: *p* = .033, Fail‐safe *N* = 3, Figure S9), and the 0.95 *omnivore* model (mist net: *p* = .032, Fail‐safe *N* = 3, Figure S10).

### 3.1 Mist‐net studies

**Figure 2:**
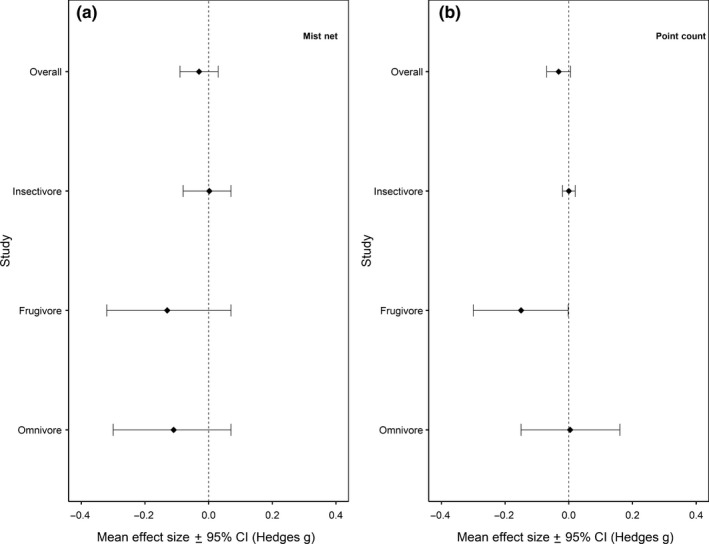
Overall effect sizes of all studies combined in each category: *Overall* (all data), *insectivore*, *frugivore*, and *omnivore*, with their respective 95% confidence intervals. Effect sizes are from the 0.75 regression quantile for (a) mist‐net studies and (b) point‐count studies

**Figure 3:**
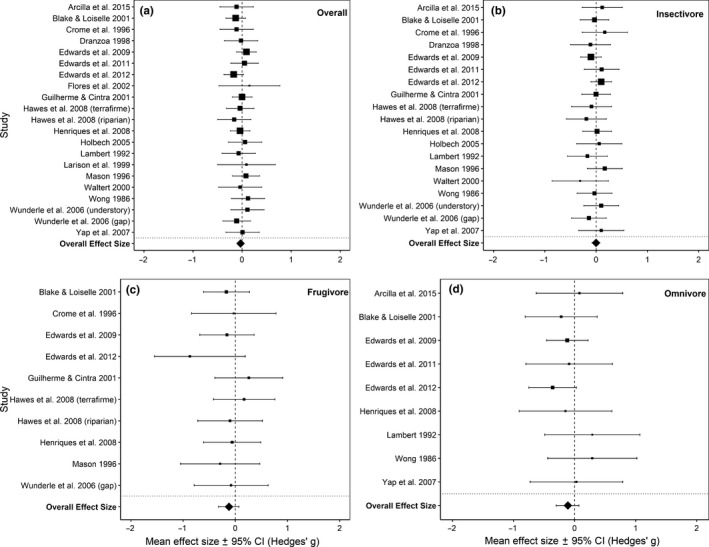
The effect sizes of each mist‐net study and the overall effect size with their respective 95% confidence intervals. The size of the points corresponds to each study's respective weights. Effect sizes are from the 0.75 regression quantile for (a) *Overall*, (b) *insectivore* foraging guild, (c) *frugivore* foraging guild, and (d) *omnivore* foraging guild

Selective logging did not affect the mass–abundance scaling of the *Overall* avian community across all regression quantiles (*p* > .05; Figures 2 and 3a, Figure S1). Confidence intervals for these effect sizes overlapped zero in all cases (Table 1), indicating considerable within‐study uncertainty in the strength of the effects of logging on mass–abundance scaling. Mass–abundance relationships did not vary significantly in relation to elevation or study continent (*p* > .05).

**Table 1:** The average Hedges' *g* effect sizes of each mass–abundance regression quantile and the 95% confidence interval for each effect size from the mist‐net data

Quantile	Average effect size	95% CI	
Overall			
0.75	−0.029	−0.090	0.032
0.80	−0.030	−0.091	0.031
0.85	−0.054	−0.115	0.007
0.90	−0.052	−0.112	0.009
0.95	−0.059	−0.120	0.002
Guild: *insectivore*			
0.75	0.002	−0.076	0.071
0.80	0.005	−0.068	0.078
0.85	0.007	−0.081	0.066
0.90	0.009	−0.064	0.083
0.95	−0.020	−0.094	0.053
Guild: *frugivore*			
0.75	−0.125	−0.315	0.066
0.80	0.001	−0.190	0.192
0.85	0.100	−0.090	0.291
0.90	0.102	−0.089	0.293
0.95	0.127	−0.065	0.318
Guild: *omnivore*			
0.75	−0.111	−0.297	0.074
0.80	−0.149	−0.334	0.037
0.85	−0.176	−0.362	0.009
**0.90**	**−0.202**	**−0.388**	**−0.017**
0.95	−0.180	−0.366	0.005

Note

Results are shown for the whole dataset (*Overall*) and for each foraging guild (*insectivore*, *frugivore*, and *omnivore*). Significant effect sizes are highlighted in bold.

Examining the effect of selective logging on the mass–abundance relationship at the *insectivore* and *frugivore* foraging guild level showed similar results across all regression quantiles (*p* > .05; Figures 2 and 3b,c, Figure S2 and S3). Again, confidence intervals for these effect sizes overlapped zero in all cases (Table 1). The effect sizes for the *insectivore* guild were also not affected by elevation and study continent (*p* > .05). However, effect sizes for the *frugivore* guild were associated with study continent in the 0.75 quantile (Asia having a significant negative effect size: mean Hedges' *g* [±95% CI] = −0.42 [−0.834, −0.009], *p* = .045) and 0.95 quantile (South America having a significant positive effect size: mean Hedges' *g* [±95% CI] = 0.53 [0.037, 1.013], *p* = .035), as well as by elevation in the 0.95 quantile where there is a significant positive effect at zero elevation (mean Hedges' *g* [±95% CI] = 0.31 [0.017, 0.606], *p* = .038). In the *omnivore* guild, the mass–abundance relationship at the 0.9 quantile became significantly more negative in selectively logged forests (mean Hedges' *g* [±95% CI] = −0.20 [−0.388, −0.017], *p* = .033; Table 1, Figure S4c) with all other quantiles showing no effect of selective logging (Table 1, Figure 3d, Figure S4). Elevation and study continent did not affect the mass–abundance scaling of the *omnivore* communities.

### 3.2 Point‐count studies

**Figure 4:**
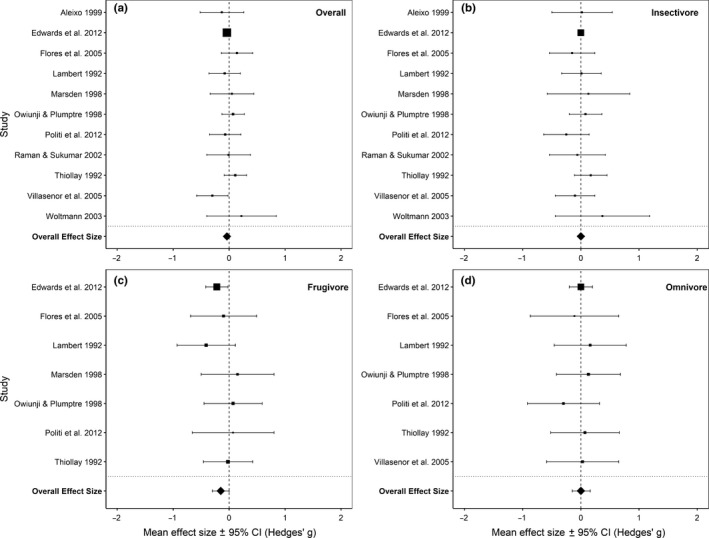
The effect sizes of each point‐count study and the overall effect size with their respective 95% confidence intervals. The size of the points corresponds to each study's respective weights. Effect sizes are from the 0.75 regression quantile for (a) *Overall*, (b) *insectivore* foraging guild, (c) *frugivore* foraging guild, and (d) *omnivore* foraging guild

There was no effect of selective logging on the mass–abundance scaling of the *Overall* bird community (Table 2, Figures 2 and 4a, Figure S5) except in the 0.95 quantile where the mass–abundance slope was significantly more negative in selectively logged forests (mean Hedges' *g* [±95% CI] = −0.05 [−0.07, −0.03], *p* < .001; Table 2, Figure S5d). When elevation and continent were both taken into account, only the African continent had a significant positive effect size (mean Hedges' *g* [±95% CI] = 0.28 [0.028, 0.540], *p* = .030) at the 0.75 quantile.

**Table 2:** The average Hedges' *g* effect sizes of each mass–abundance regression quantile and the 95% confidence interval for each effect size from the point‐count data

Quantile	Average effect size	95% CI	
Overall			
0.75	−0.032	−0.070	0.005
0.80	−0.010	−0.029	0.009
0.85	−0.011	−0.063	0.042
0.90	0.000	−0.020	0.019
**0.95**	**−0.05**	**−0.070**	**−0.030**
Guild: *insectivore*			
0.75	0.000	−0.019	0.020
0.80	0.009	−0.028	0.011
0.85	0.001	−0.019	0.020
0.90	0.010	−0.033	0.052
0.95	0.001	−0.018	0.021
Guild: *frugivore*			
**0.75**	**−0.150**	**−0.299**	**−0.002**
**0.80**	**−0.172**	**−0.321**	**−0.024**
0.85	−0.007	−0.156	0.141
0.90	−0.112	−0.261	0.037
**0.95**	**−0.165**	**−0.313**	**−0.016**
Guild: *omnivore*			
0.75	0.004	−0.152	0.159
0.80	0.028	−0.127	0.184
0.85	−0.029	−0.285	0.227
0.90	−0.070	−0.301	0.160
0.95	−0.025	−0.299	0.249

Note

Results are shown for the whole dataset (*Overall*) and for each foraging guild (*insectivore*, *frugivore*, and *omnivore*). Significant effect sizes are highlighted in bold.

At the foraging guild level, the mass–abundance relationships of the *insectivore* and *omnivore* communities were unaffected by selective logging (*p* > .05; Table 2, Figure 2, *insectivore*: Figure 4b, Figure S6, *omnivore*: Figure 4d, Figure S8) and there was no influence of elevation and study continent (*p* > .05). Contrarily, the mass–abundance scaling of *frugivore* was significantly more negative in selectively logged forests in the 0.75 (mean Hedges' *g* [±95% CI] = −0.15 [−0.299, −0.002], *p* = .047; Figure 2), 0.8 (mean Hedges' *g* [±95% CI] = −0.17 [−0.321, −0.024], *p* = .023), and 0.95 (mean Hedges' *g* [±95% CI] = −0.17 [−0.313, −0.016], *p* = .030) quantiles (Table 2, Figure 4c, Figure S7). However, elevation and study continent did not affect these mass–abundance relationships (*p* > .05).

## 4 DISCUSSION

We investigated how selective logging affects the mass–abundance relationship in avian communities across the tropics, finding that communities sampled by mist netting largely experienced no effect of selective logging on these relationships, except in the *omnivore* communities, where the mass–abundance relationship became more negative in selectively logged forests. On the other hand, we found that the mass–abundance relationship of the overall communities sampled by point counts was more negative in logged forests. This was likely driven by the *frugivore* communities, which were the only foraging guild to have a more negative mass–abundance relationship in selectively logged forests. This increased negative slope in the mass–abundance relationship could indicate a loss of larger species or a rise in the number of small species (Srinivasan, 2013), potentially signaling changes in resource and energy partitioning between species, with downstream impacts on ecosystem functioning.

Srinivasan (2013) found that the mass–abundance relationship of understory avian insectivores became steeper and more negative as logging intensity increased. This was thought to be due to multiple factors such as resource depletion and density compensation. As resources decline in degraded habitats, disproportionately vulnerable larger species may decrease in abundance and thus free up resources for smaller species to thus increase in abundance. Hunting could also be a factor leading to declines in larger species, as selectively logged forests tend to have increased hunting pressure due to more accessibility via logging roads (Sheil & Meijaard, 2005).

Different functional groups consume energy at different rates and have different amounts of energy available to them in their habitat (Ernest et al., 2003; Marquet, 2002) and although species which forage on fruits and nectar (*frugivore* guild) tend to thrive in degraded forests compared with species which forage on invertebrates (*insectivore* guild; Greenberg, Bichier, & Sterling, 1997; Sreekar et al., 2015), we observe that the mass–abundance relationship of *frugivore* point‐count communities were affected by selective logging and not the *insectivore* communities. This could indicate greater vulnerability of larger frugivore species, perhaps because large fruiting trees tend to be removed during the logging process and require a longer period of time to regenerate (Burivalova et al., 2015). Furthermore, some large frugivore species, such as the Helmeted Hornbill (*Rhinoplax vigil*), are particularly threatened by hunting due to their value for meat, ornamental feathers or ivory‐like casque (Beastall, Shepherd, Hadiprakarsa, & Martyr, 2016; Bennett, Nyaoi, & Sompud, 1997). The change in the mass–abundance relationship in these frugivore communities could precipitate important changes in seed dispersal services within logged forest ecosystems.

On the other hand, our results show that many mass–abundance relationships in the mist‐net and point‐count communities are robust to selective logging activities. Many studies from freshwater, intertidal, and marine ecosystems have shown that the mass–abundance relationship is robust to disturbances (Jonsson, Cohen, & Carpenter, 2005; Marquet, Navarrete, & Castilla, 1990; O'Gorman & Emmerson, 2011). O'Gorman and Emmerson (2011) found that the mass–abundance relationship in marine food webs was robust to disturbances due to higher species turnover in the disturbed communities. One potential mechanism is structural changes in habitat or food webs produce new size–abundance niches that could be exploited by new species. Similarly, there could be a replacement of species with similar body sizes after logging, allowing the community to maintain energy and structural stability within the system (Damuth, 1981, 1987; Jonsson et al., 2005; Marquet et al., 1990).

Sreekar et al. (2015) also found no difference in the mass–abundance relationship of avian communities between primary forests and selectively logged forests in southern India. Sreekar et al. (2015) suggested that this similarity was due to high species turnover in logged forests, as the avian communities change in response to altered environments. They observed a higher proportion of insectivores in primary forests compared with logged forests and a higher proportion of insectivores in the understory primary forest community compared with the logged community. Given that there is a limited amount of energy available within a logged habitat, it is perhaps unsurprising that mass–abundance relationships are not consistently affected as communities adapt to the amount of available energy. Nevertheless, marked changes in avian community structure following logging suggest that other ecosystem properties in logged forests may be different from that of primary forests, representing an important topic for future studies. These different responses found between Srinivasan (2013) and Sreekar et al. (2015) may be due to the degree of habitat variation. For instance, Srinivasan (2013) sampled across a gradient of selective logging intensities, while this study and Sreekar et al. (2015) sampled across a gradient of distinct habitats which are more drastically different from each other. In the two distinct habitats, the logged forest communities could have had time to reach a new state of energy and structural equilibrium that still adheres to the power law $N\propto M^{b}$, which describes the mass–abundance relationship, where *N* is species abundance and *M* is the species' body mass. Thus, both logged and primary forests in this case would have similar mass–abundance slopes.

### 4.1 Synthesis and applications

Our study suggests that logging only significantly alters the avian mass–abundance relationships of selected *frugivore* and *omnivore* communities in tropical forests. Inclusion of point‐count studies in the meta‐analysis was important as these detected some changes in the mass‐abundance relationship that the mist‐net studies failed to capture. The lack of impacts on understory communities in the mist‐net studies may be due to mist nets only detecting a subset of bird sizes, making it difficult to detect abundance changes in species at the upper extreme of the size spectrum.

The impacts experienced by these communities could be minimized by restoring selectively logged forests with native fruiting trees, especially those bearing larger fruits. The results also show that logging does not change the mass–abundance relationship of the majority of the avian communities which adds weight to the evidence that avian communities are relatively robust to selective logging, with species and communities exhibiting some flexibility to adapt to modified environments and, in doing so, maintaining ecosystem functioning (Ewers et al., 2015), which is crucial in an epoch of global change. These results also underscore the high conservation value of logged tropical forests (Edwards et al., 2011), indicating that an urgent conservation priority is the protection of these cost‐effective habitats from further degradation and deforestation, allowing enhanced area of forest protection, buffering of primary forest reserves and maintenance of landscape‐scale connectivity (Edwards, Tobias, et al., 2014).

## CONFLICT OF INTEREST

None declared.

## AUTHORS' CONTRIBUTIONS

US, DPE, CCPC, and JJG conceived the ideas and designed the methodology; US, MGH, and CCPC collected the data; and CCPC analyzed the data and led the writing. All authors contributed critically to the drafts and gave final approval for publication.

## Data Availability

Data available from Figshare: https://doi.org/10.15131/shef.data.11590902.v1
