# Supplementary material for: Mass–abundance scaling in avian communities is maintained after tropical selective logging
Source: Ecol Evol. 2020 Feb 29;10(6):2803–12. doi: 10.1002/ece3.6066 (PMC7083669; doi:10.1002/ece3.6066)

**Supplementary materials and methods**

**Mass-abundance scaling in avian communities is maintained after tropical selective logging**

**Appendix A**

**Table S1** Information about all mist-net studies and the proportion of guilds in each study

**Table S2** Information about all point-count studies and the proportion of guilds in each study.

**Table S3** Information about the foraging guild classes assigned to each species.

**Fig. S1** Effect sizes from the 0.8, 0.85, 0.9 and 0.95 regression quantiles for *Overall* birds in mist-netting studies.

**Fig. S2** Effect sizes from the 0.8, 0.85, 0.9 and 0.95 regression quantiles for *Insectivore* foraging guild in mist-netting studies.

**Fig. S3** Effect sizes from the 0.8, 0.85, 0.9 and 0.95 regression quantiles for *Frugivore* foraging guild in mist-netting studies.

**Fig. S4** Effect sizes from the 0.8, 0.85, 0.9 and 0.95 regression quantiles for *Omnivore* foraging guild in mist-netting studies.

**Fig. S5** Effect sizes from the 0.8, 0.85, 0.9 and 0.95 regression quantiles for *Overall* birds in point-count studies.

**Fig. S6** Effect sizes from the 0.8, 0.85, 0.9 and 0.95 regression quantiles for *Insectivore* foraging guild in point-count studies.

**Fig. S7** Effect sizes from the 0.8, 0.85, 0.9 and 0.95 regression quantiles for *Frugivore* foraging guild in point-count studies.

**Fig. S8** Effect sizes from the 0.8, 0.85, 0.9 and 0.95 regression quantiles for *Omnivore* foraging guild in point-count studies.

**Fig. S9** Funnel plots for Funnel plots of effect sizes for each mist-net study.

**Fig. S10** Funnel plots for Funnel plots of effect sizes for each point-count study.

TABLE S1 Information of all mist-net studies including continent where the study was conducted, type of logging conducted in the study sites and the proportion of each guild in each study.

| **Study** | **Continent** | **Logging**  **type** | **Guild proportions** | |
| --- | --- | --- | --- | --- |
|  |  |  | **Foraging Guild** | **Proportion** |
| Arcilla N, Holbech LH, O’Donnell S. 2015. Severe declines of understory birds following illegal logging in Upper Guinea forests of Ghana, West Africa. Biological Conservation 188:41-49. | Africa | - Conventional logging - Salvage logging | Insectivore | 0.68 |
|  |  |  | Frugivore | 0.10 |
|  |  |  | Omnivore | 0.20 |
|  |  |  | Carnivore | 0.02 |
|  |  |  | Granivore | 0.00 |
| Blake JG, Loiselle BA. 2001. Bird assemblages in second-growth and old-growth forests, Costa Rica: Perspectives from mist nets and point counts. Auk 118:304-326. | Central America | - Conventional logging | Insectivore | 0.57 |
|  |  |  | Frugivore | 0.24 |
|  |  |  | Omnivore | 0.13 |
|  |  |  | Carnivore | 0.03 |
|  |  |  | Granivore | 0.03 |
| Crome FHJ, Thomas MR, Moore LA. 1996. A novel Bayesian approach to assessing impacts of rain forest logging. Ecological Applications 6:1104-1123. | Australia | - Conventional logging | Insectivore | 0.66 |
|  |  |  | Frugivore | 0.19 |
|  |  |  | Omnivore | 0.14 |
|  |  |  | Carnivore | 0.02 |
|  |  |  | Granivore | 0.00 |
| Dranzoa C. 1998. The avifauna 23 years after logging in Kibale National park, Uganda. Biodiversity and Conservation 7:777-797. | Africa | - Conventional logging | Insectivore | 0.64 |
|  |  |  | Frugivore | 0.13 |
|  |  |  | Omnivore | 0.14 |
|  |  |  | Carnivore | 0.01 |
|  |  |  | Granivore | 0.09 |
| Edwards DP, Ansell FA, Ahmad AH, Nilus R, Hamer KC. 2009. The Value of Rehabilitating Logged Rainforest for Birds. Conservation Biology 23:1628–1633. | Asia | - Conventional logging | Insectivore | 0.68 |
|  |  |  | Frugivore | 0.08 |
|  |  |  | Omnivore | 0.23 |
|  |  |  | Carnivore | 0.01 |
|  |  |  | Granivore | 0.00 |
| Edwards DP, Larsen TH, Docherty TDS, Ansell FA, Hsu WW, Derhe MA, Hamer KC, Wilcove DS. 2011. Degraded lands worth protecting: the biological importance of Southeast Asia's repeatedly logged forests. Proceedings of the Royal Society B-Biological Sciences 278:82-90. | Asia | - Conventional logging | Insectivore | 0.71 |
|  |  |  | Frugivore | 0.09 |
|  |  |  | Omnivore | 0.17 |
|  |  |  | Carnivore | 0.02 |
|  |  |  | Granivore | 0.02 |
| Edwards DP, Woodcock P, Edwards FA, Larsen TH, Hsu WW, Benedick S, Wilcove S. 2012. Reduced-impact logging and biodiversity conservation: a case study from Borneo. Ecological Applications 22:561–571. | Asia | - Conventional logging - Reduced-impact logging | Insectivore | 0.69 |
|  |  |  | Frugivore | 0.08 |
|  |  |  | Omnivore | 0.21 |
|  |  |  | Carnivore | 0.01 |
|  |  |  | Granivore | 0.01 |
| Flores B, Rumiz DI, Fredericksen TS, Fredericksen NJ. 2002. El uso de claros de aprovechamiento forestal por la avifauna de un bosque semideciduo chiquitano de Santa Cruz, Bolivia. Hornero 17: 61-69. | South America | - Conventional logging | Insectivore | 0.66 |
|  |  |  | Frugivore | 0.26 |
|  |  |  | Omnivore | 0.06 |
|  |  |  | Carnivore | 0.00 |
|  |  |  | Granivore | 0.02 |
| Guilherme E, Cintra R. 2001. Effects of intensity and age of selective logging and tree girdling on an understory bird community composition in Central Amazonia, Brazil. Ecotropica 7:77-92. | South America | - Conventional logging | Insectivore | 0.79 |
|  |  |  | Frugivore | 0.15 |
|  |  |  | Omnivore | 0.05 |
|  |  |  | Carnivore | 0.01 |
|  |  |  | Granivore | 0.00 |
| Hawes J, Barlow J, Gardner TA, Peres CA. 2008. The value of forest strips for understorey birds in an Amazonian plantation landscape. Biological Conservation 141:2262-2278. | South America | - Conventional logging | Insectivore | 0.60 |
|  |  |  | Frugivore | 0.26 |
|  |  |  | Omnivore | 0.09 |
|  |  |  | Carnivore | 0.02 |
|  |  |  | Granivore | 0.03 |
| Henriques LMP, Wunderle Jr. JM, Oren DC, Willig MR. 2008. Efeitos da exploração madeireira de baixo impacto sobre uma comunidade de aves de sub-bosque na Floresta Nacional do Tapajós, Pará, Brasil. Acta Amazonica 38:267-290. | South America | - Conventional logging | Insectivore | 0.74 |
|  |  |  | Frugivore | 0.14 |
|  |  |  | Omnivore | 0.07 |
|  |  |  | Carnivore | 0.03 |
|  |  |  | Granivore | 0.01 |
| Holbech LH. 2005. The implications of selective logging and forest fragmentation for the conservation of avian diversity in evergreen forests of south-west Ghana. Bird Conservation International 15:27-52. | Africa | - Conventional logging - Salvage logging | Insectivore | 0.71 |
|  |  |  | Frugivore | 0.09 |
|  |  |  | Omnivore | 0.17 |
|  |  |  | Carnivore | 0.02 |
|  |  |  | Granivore | 0.01 |
| Lambert FR. 1992. The Consequences of Selective Logging for Bornean Lowland Forest Birds. Phil. Trans. R. Soc. Lond. B. 335:443-457. | Asia | - Conventional logging | Insectivore | 0.71 |
|  |  |  | Frugivore | 0.11 |
|  |  |  | Omnivore | 0.18 |
|  |  |  | Carnivore | 0.00 |
|  |  |  | Granivore | 0.00 |
| Larison B, et al. 1999. Biotic Surveys of Bioko and Rio Muni, Equatorial Guinea, Biodiversity Support Program, USA. | Africa | - Conventional logging | Insectivore | 0.67 |
|  |  |  | Frugivore | 0.14 |
|  |  |  | Omnivore | 0.14 |
|  |  |  | Carnivore | 0.02 |
|  |  |  | Granivore | 0.03 |
| Mason D. 1996. Responses of venezuelan understory birds to selective logging, enrichment strips, and vine cutting. Biotropica 28:296-309. | South America | - Conventional logging | Insectivore | 0.74 |
|  |  |  | Frugivore | 0.14 |
|  |  |  | Omnivore | 0.07 |
|  |  |  | Carnivore | 0.03 |
|  |  |  | Granivore | 0.01 |
| Waltert M. 2000. Forest management and the distribution of understorey birds in the Bossematié Forest, eastern Ivory Coast. Ostrich 71:295-299. | Africa | - Conventional logging | Insectivore | 0.68 |
|  |  |  | Frugivore | 0.08 |
|  |  |  | Omnivore | 0.18 |
|  |  |  | Carnivore | 0.01 |
|  |  |  | Granivore | 0.04 |
| Wong M. 1986. Trophic organization of understory birds in a Malaysian dipterocarp forest. Auk 103:100-116. | Asia | - Conventional logging | Insectivore | 0.74 |
|  |  |  | Frugivore | 0.07 |
|  |  |  | Omnivore | 0.17 |
|  |  |  | Carnivore | 0.01 |
|  |  |  | Granivore | 0.01 |
| Wunderle JM, Henriques LMP, Willig MR. 2006. Short-term responses of birds to forest gaps and understory: An assessment of reduced-impact logging in a lowland Amazon forest. Biotropica 38:235-255. | South America | - Conventional logging - Reduced-impact logging | Insectivore | 0.75 |
|  |  |  | Frugivore | 0.15 |
|  |  |  | Omnivore | 0.06 |
|  |  |  | Carnivore | 0.03 |
|  |  |  | Granivore | 0.01 |
| Yap CAM, Sodhi NS, Peh KSH. 2007. Phenology of tropical birds in Peninsular Malaysia: Effects of selective logging and food resources. Auk 124:945-961. | Asia | - Conventional logging | Insectivore | 0.67 |
|  |  |  | Frugivore | 0.09 |
|  |  |  | Omnivore | 0.22 |
|  |  |  | Carnivore | 0.03 |
|  |  |  | Granivore | 0.00 |

TABLE S2 Information of all point-count studies including continent where the study was conducted, type of logging conducted in the study sites and the proportion of each guild in each study.

| **Study** | **Continent** | **Logging type** | **Guild proportions** | |
| --- | --- | --- | --- | --- |
|  |  |  | **Foraging Guild** | **Proportion** |
| Aleixo A. 1999. Effects of selective logging on a the Brazilian Atlantic bird community in forest. The Condor **101**:537-548. | South America | - Conventional logging | Insectivore | 0.53 |
|  |  |  | Frugivore | 0.21 |
|  |  |  | Omnivore | 0.16 |
|  |  |  | Carnivore | 0.04 |
|  |  |  | Granivore | 0.07 |
| Edwards DP, Woodcock P, Edwards FA, Larsen TH, Hsu WW, Benedick S, Wilcove S. 2012. Reduced-impact logging and biodiversity conservation: a case study from Borneo. Ecological Applications **22**:561–571. | Asia | - Conventional logging | Insectivore | 0.66 |
|  |  |  | Frugivore | 0.16 |
|  |  |  | Omnivore | 0.17 |
|  |  |  | Carnivore | 0.01 |
|  |  |  | Granivore | 0.00 |
| Flores B, Rumiz DI, Blate GM. 2005. Estructura de la vegetación y de la comunidad de aves en un bosque intervenido de la Chonta, Guarayos, Santa Cruz. Rev. Bol. Ecol. **18**: 33- 50. | South America | - Conventional logging | Insectivore | 0.53 |
|  |  |  | Frugivore | 0.26 |
|  |  |  | Omnivore | 0.14 |
|  |  |  | Carnivore | 0.01 |
|  |  |  | Granivore | 0.05 |
| Lambert FR. 1992. The Consequences of Selective Logging for Bornean Lowland Forest Birds. Phil. Trans. R. Soc. Lond. B. **335**:443-457. | Asia | - Conventional logging | Insectivore | 0.60 |
|  |  |  | Frugivore | 0.23 |
|  |  |  | Omnivore | 0.15 |
|  |  |  | Carnivore | 0.01 |
|  |  |  | Granivore | 0.01 |
| Marsden SJ. 1998. Changes in Bird Abundance Following Selective Logging on Seram, Indonesia. Conservation Biology **12**:605-611. | Asia | - Conventional logging | Insectivore | 0.32 |
|  |  |  | Frugivore | 0.37 |
|  |  |  | Omnivore | 0.16 |
|  |  |  | Carnivore | 0.05 |
|  |  |  | Granivore | 0.10 |
| Owiunji I, Plumptre AJ. 1998. Bird communities in logged and unlogged compartments in Budongo Forest, Uganda. Forest Ecology and Management **108**:115–126. | Africa | - Conventional logging | Insectivore | 0.61 |
|  |  |  | Frugivore | 0.19 |
|  |  |  | Omnivore | 0.16 |
|  |  |  | Carnivore | 0.01 |
|  |  |  | Granivore | 0.04 |
| Politi N, Hunter Jr M, Rivera L. 2012. Assessing the effects of selective logging on birds in Neotropical piedmont and cloud montane forests. Biodiversity Conservation **21**:3131-3155. | South America | - Conventional logging | Insectivore | 0.57 |
|  |  |  | Frugivore | 0.14 |
|  |  |  | Omnivore | 0.21 |
|  |  |  | Carnivore | 0.02 |
|  |  |  | Granivore | 0.06 |
| Raman TRS, Sukumar R. 2002. Responses of tropical rainforest birds to abandoned plantations, edges and logged forest in the Western Ghats, India. Animal Conservation **5**:201–216. | Asia | - Conventional logging | Insectivore | 0.64 |
|  |  |  | Frugivore | 0.12 |
|  |  |  | Omnivore | 0.22 |
|  |  |  | Carnivore | 0.01 |
|  |  |  | Granivore | 0.00 |
| Thiollay JM. 1992. Influence of Selective Logging on Bird Species Diversity in a Guianan Rain Forest. Conservation Biology **6**:47-63. | South America | - Conventional logging | Insectivore | 0.57 |
|  |  |  | Frugivore | 0.25 |
|  |  |  | Omnivore | 0.14 |
|  |  |  | Carnivore | 0.00 |
|  |  |  | Granivore | 0.04 |
| Villaseñor JF, Sosa N, Villaseñor L. 2005. Effects of Selective Logging on Birds in the Sierra de Coalcomán, Sierra Madre del Sur, Michoacán, Western Mexico. USDA Forest Service Gen. Tech. Rep. PSW-GTR-191. 381-390. | South America | - Conventional logging | Insectivore | 0.62 |
|  |  |  | Frugivore | 0.10 |
|  |  |  | Omnivore | 0.18 |
|  |  |  | Carnivore | 0.02 |
|  |  |  | Granivore | 0.09 |
| Woltmann S. 2003. Bird community responses to disturbance in a forestry concession in lowland Bolivia. Biodiversity and Conservation **12**:1921–1936. | South America | - Conventional logging | Insectivore | 0.62 |
|  |  |  | Frugivore | 0.24 |
|  |  |  | Omnivore | 0.14 |
|  |  |  | Carnivore | 0.00 |
|  |  |  | Granivore | 0.00 |

TABLE S3 Information about the foraging guild classes assigned to each species. The categorisation of each foraging guild is based on the EltonTraits 1.0 database (Wilman et al. 2014).

| **Foraging Guild** | **EltonTraits 1.0 Category** | **Diet** |
| --- | --- | --- |
|  |  |  |
| Insectivore | Invertebrate | Invertebrates |
| Frugivore | FruiNect | Fruits and Nectar |
| Omnivore | Omnivore | Consisting of less than or equal to 50% of all four categories (Invertebrate, FruitNect, VertFishScav, PlantSeed) |
| Carnivore | VertFishScav | Vertebrates, Fish and Carrion |
| Granivore | PlantSeed | Plants and Seeds |


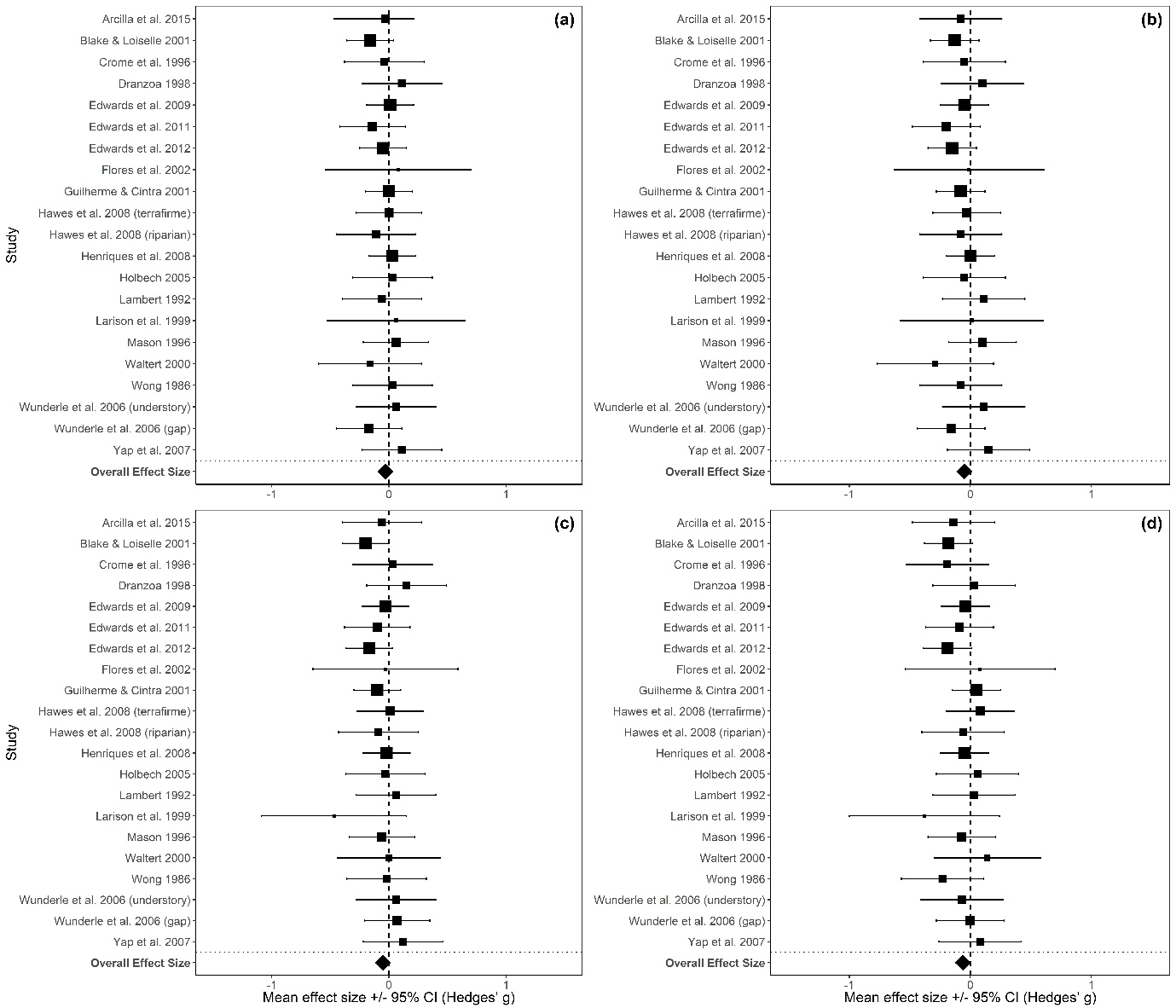
**Fig. S1** The effect sizes of each mist-net study from the *Overall* analysis and the overall effect size with their respective 95% confidence intervals. The size of the points corresponds to each study’s respective weights. Effect sizes are from the (**a**) 0.80, (**b**) 0.85, (**c**) 0.90 and (**d**) 0.95 regression quantile.

**Fig. S2** The effect sizes of each mist-net study from the *Insectivore* analysis and the overall effect size with their respective 95% confidence intervals. The size of the points corresponds to each study’s respective weights. Effect sizes are from the (**a**) 0.80, (**b**) 0.85, (**c**) 0.90 and (**d**) 0.95 regression quantile.


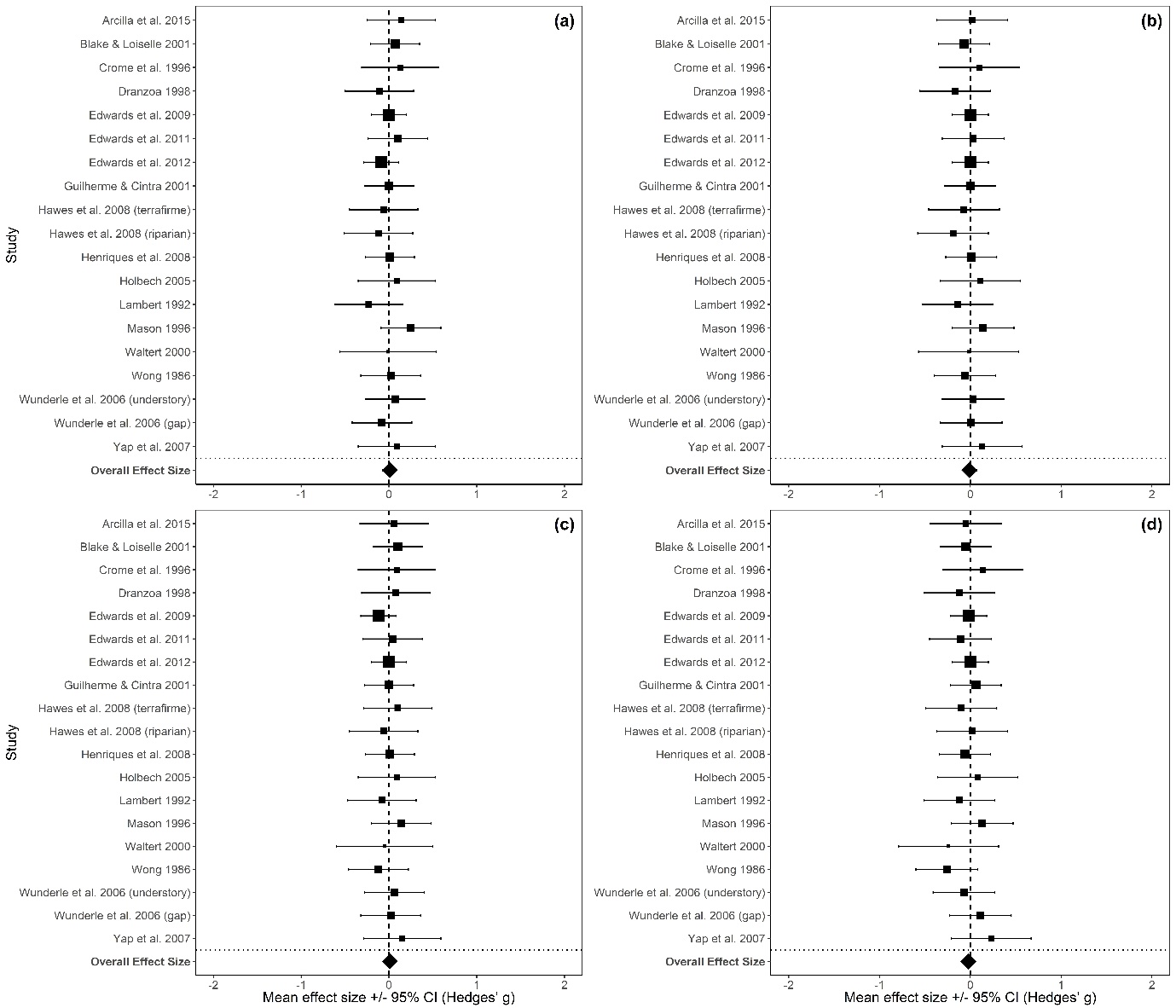


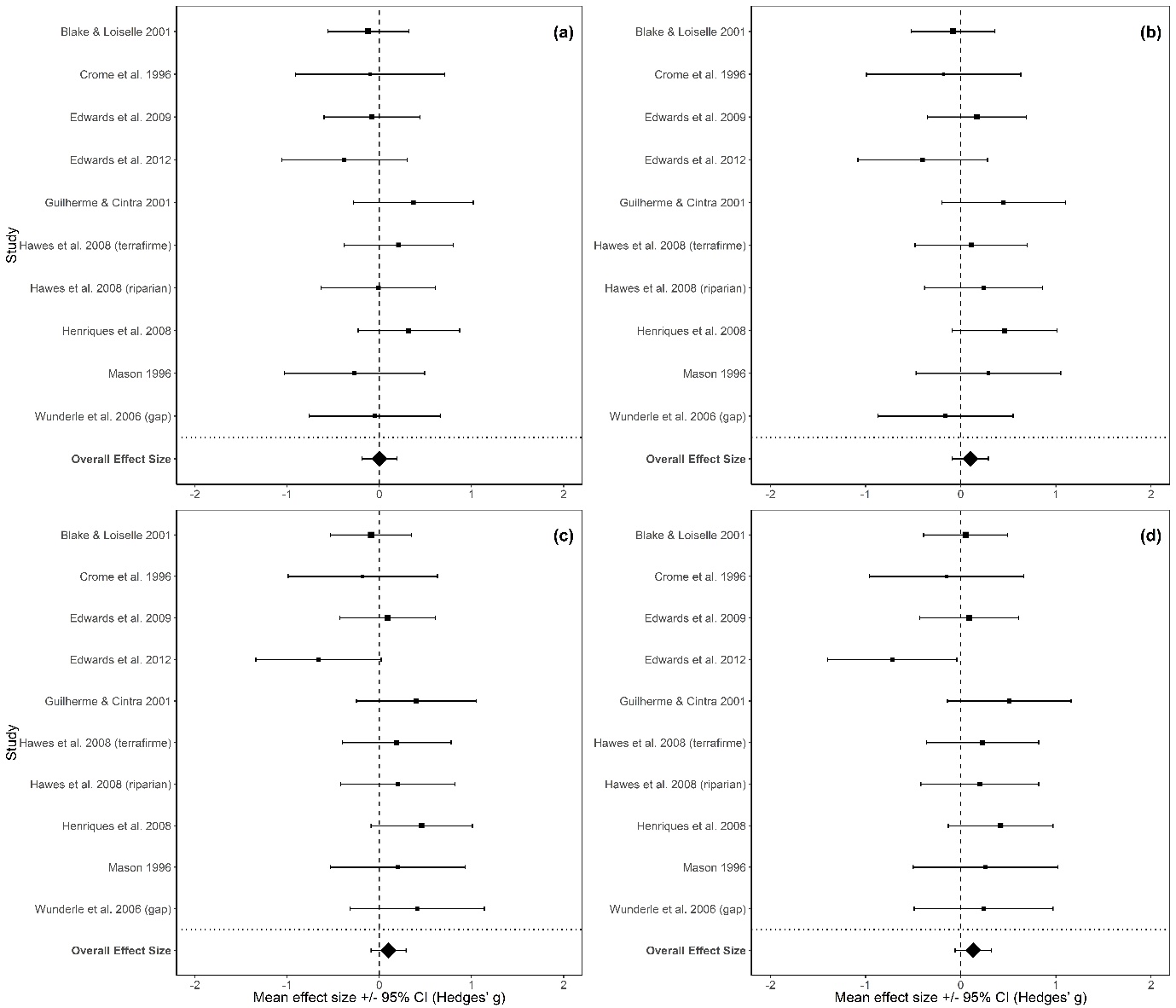
**Fig. S3** The effect sizes of each mist-net study from the *Frugivore* analysis and the overall effect size with their respective 95% confidence intervals. The size of the points corresponds to each study’s respective weights. Effect sizes are from the (**a**) 0.80, (**b**) 0.85, (**c**) 0.90 and (**d**) 0.95 regression quantile.


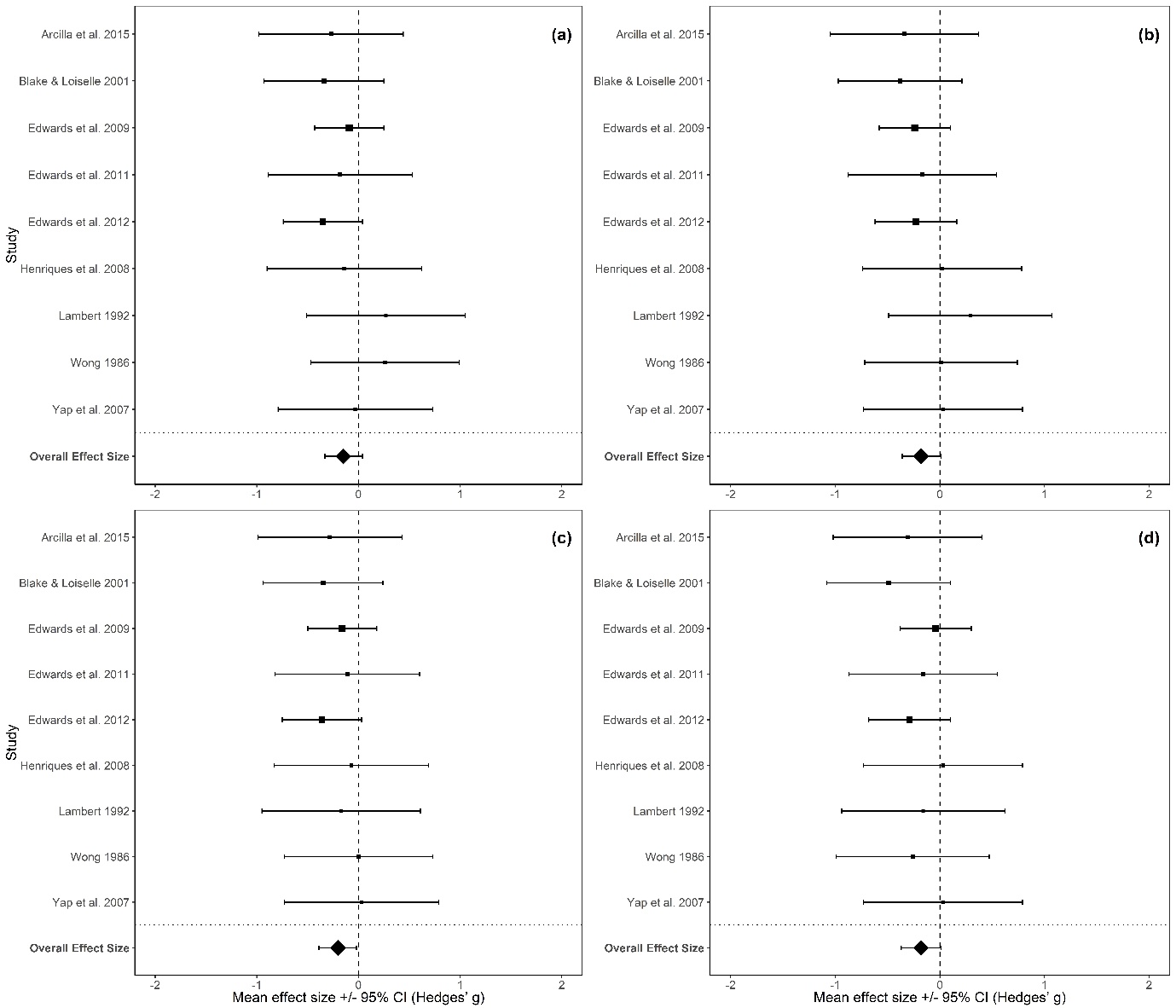
**Fig. S4** The effect sizes of each mist-net study from the *Omnivore* analysis and the overall effect size with their respective 95% confidence intervals. The size of the points corresponds to each study’s respective weights. Effect sizes are from the (**a**) 0.80, (**b**) 0.85, (**c**) 0.90 and (**d**) 0.95 regression quantile.


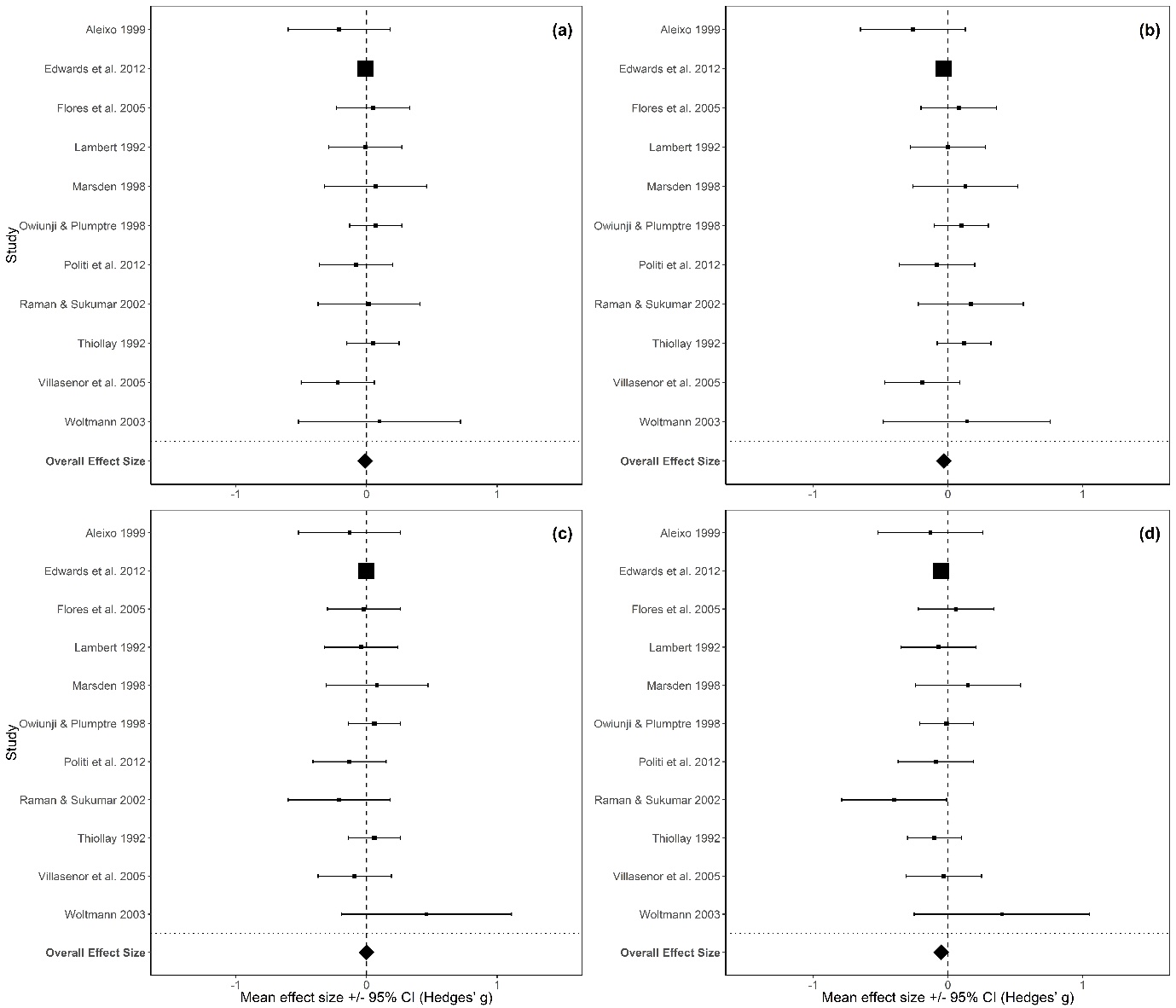
**Fig. S5** The effect sizes of each point-count study from the *Overall* analysis and the overall effect size with their respective 95% confidence intervals. The size of the points corresponds to each study’s respective weights. Effect sizes are from the (**a**) 0.80, (**b**) 0.85, (**c**) 0.90 and (**d**) 0.95 regression quantile.

**Fig. S6** The effect sizes of each point-count study from the *Insectivore* analysis and the overall effect size with their respective 95% confidence intervals. The size of the points corresponds to each study’s respective weights. Effect sizes are from the (**a**) 0.80, (**b**) 0.85, (**c**) 0.90 and (**d**) 0.95 regression quantile.


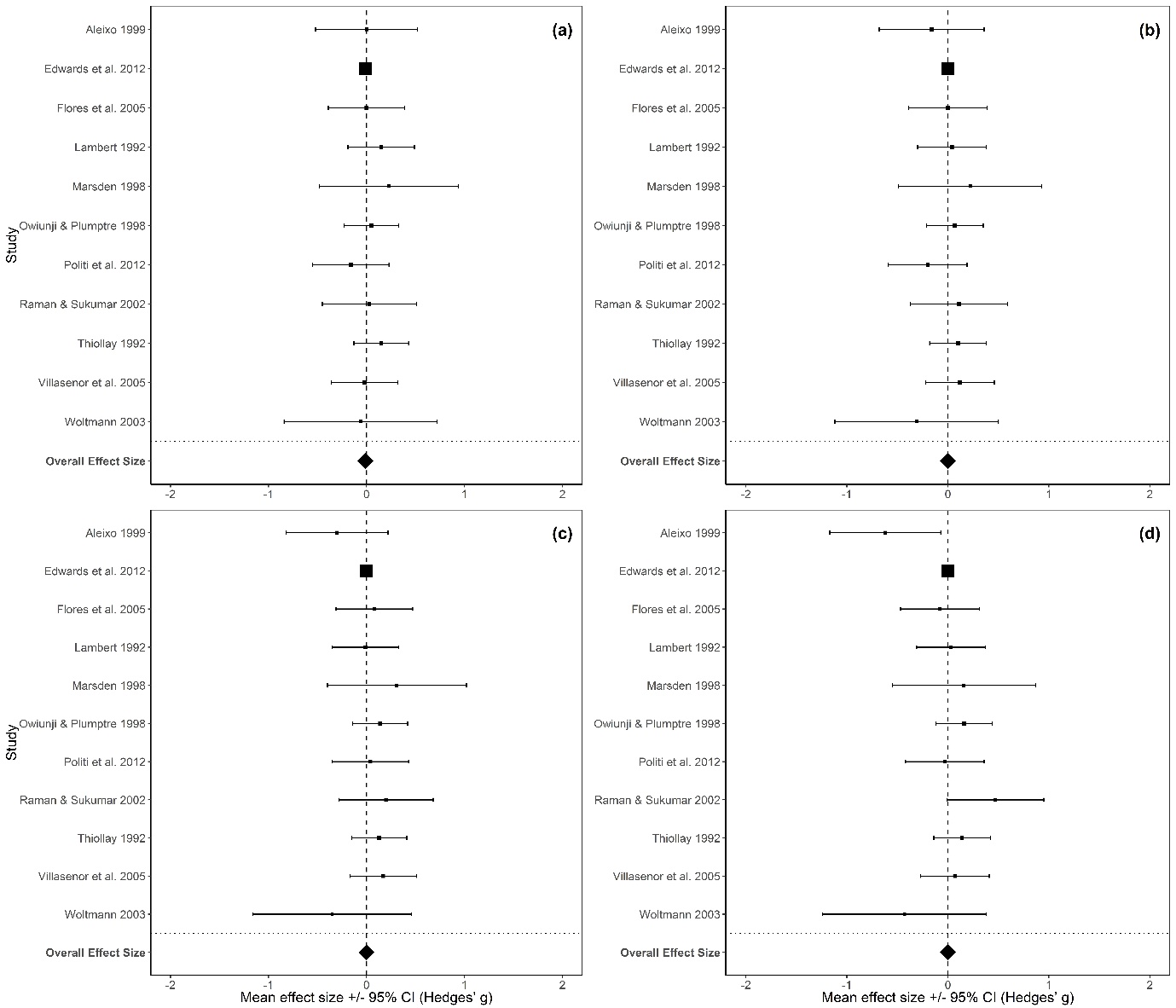


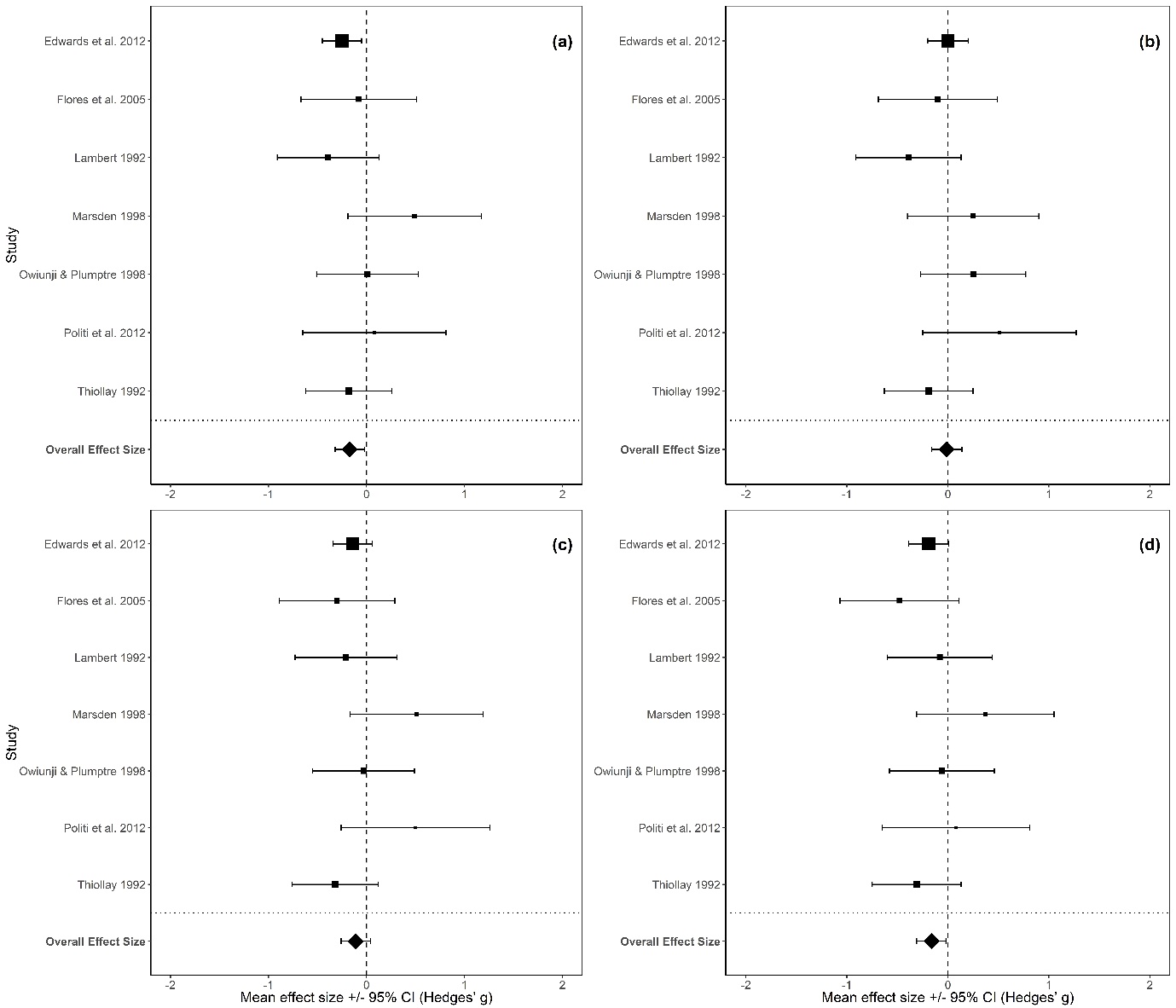
**Fig. S7** The effect sizes of each point-count study from the *Frugivore* analysis and the overall effect size with their respective 95% confidence intervals. The size of the points corresponds to each study’s respective weights. Effect sizes are from the (**a**) 0.80, (**b**) 0.85, (**c**) 0.90 and (**d**) 0.95 regression quantile.


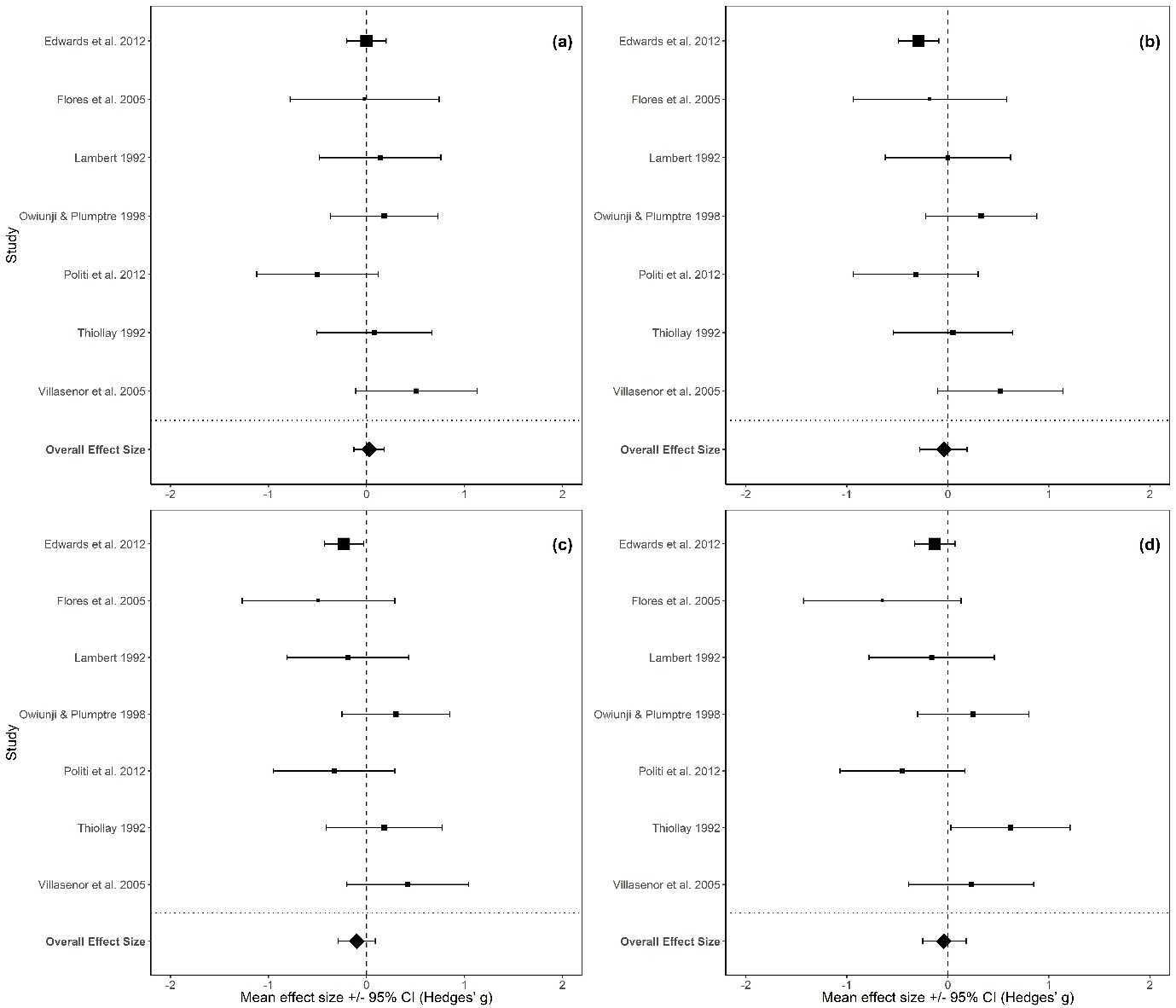
**Fig. S8** The effect sizes of each point-count study from the *Omnivore* analysis and the overall effect size with their respective 95% confidence intervals. The size of the points corresponds to each study’s respective weights. Effect sizes are from the (**a**) 0.80, (**b**) 0.85, (**c**) 0.90 and (**d**) 0.95 regression quantile.

**Fig. S9** Funnel plots of effect sizes (0.75, 0.8, 0.85, 0.9, 0.95 quantiles) for each mist-net study *Overall* and foraging guilds (*Insectivore*, *Frugivore* and *Omnivore*).


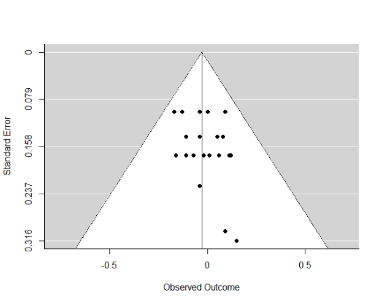


**OVERALL**

**INSECTIVORE**

**OMNIVORE**

**FRUGIVORE**

**0.75**

**0.95**

**0.90**

**0.85**

**0.80**


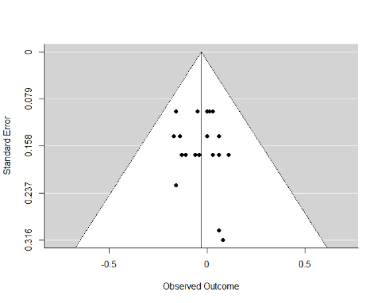

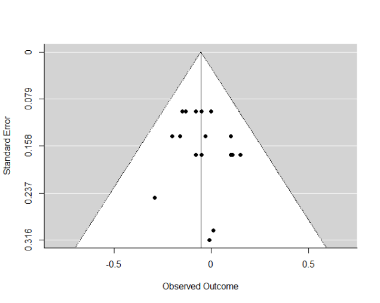

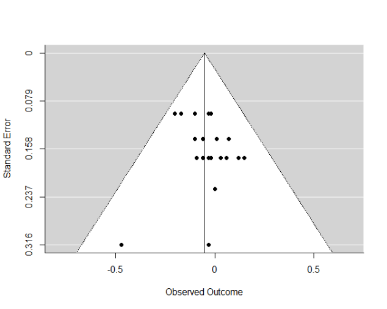

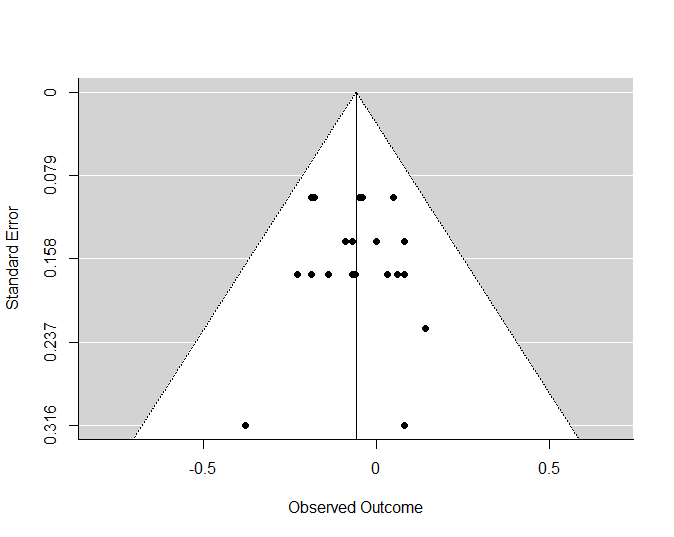

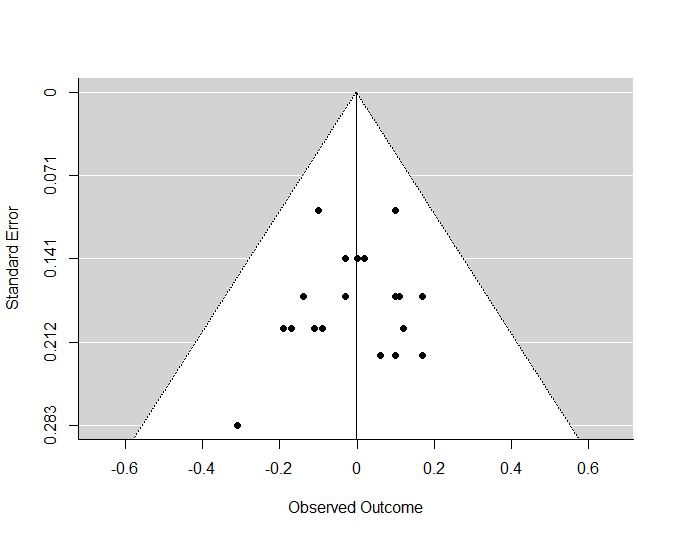

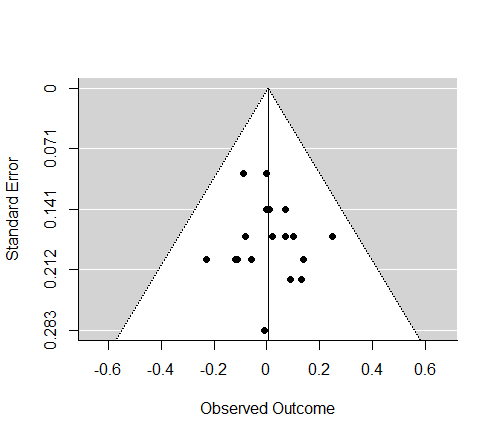

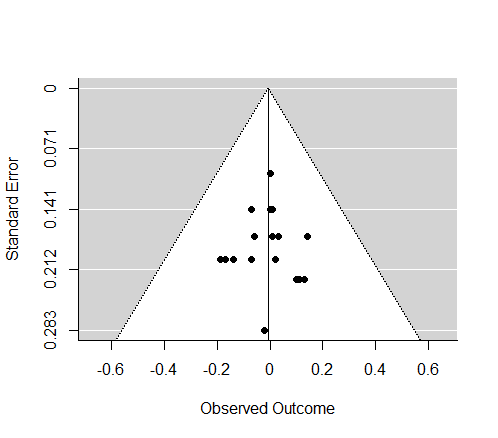

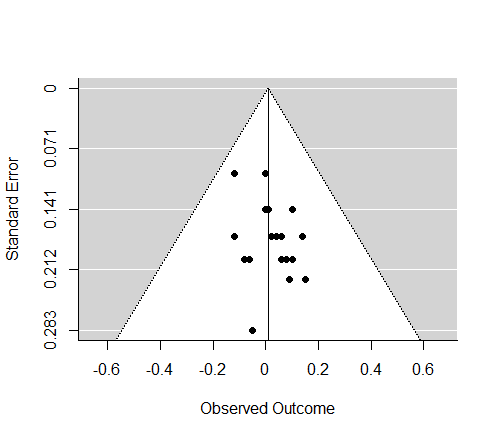

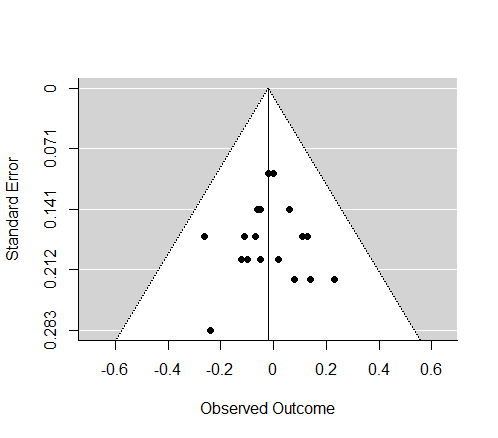

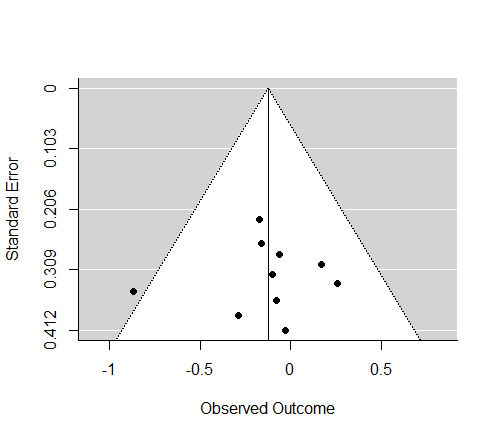

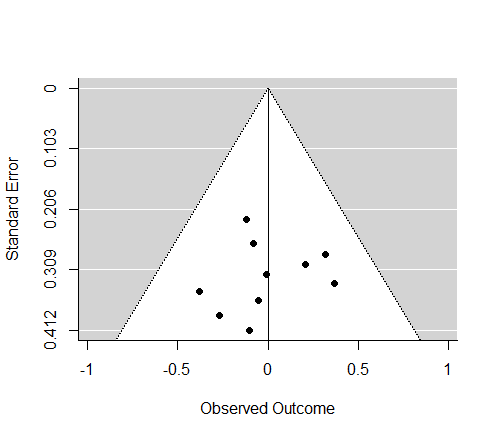

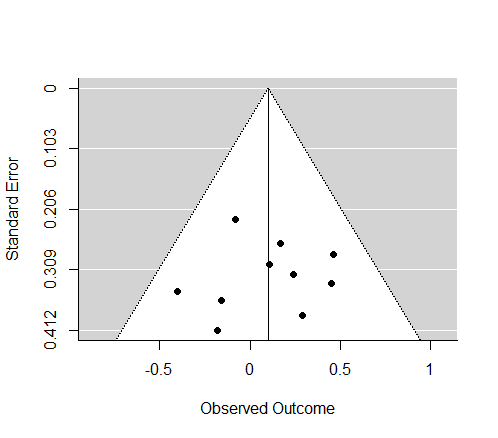

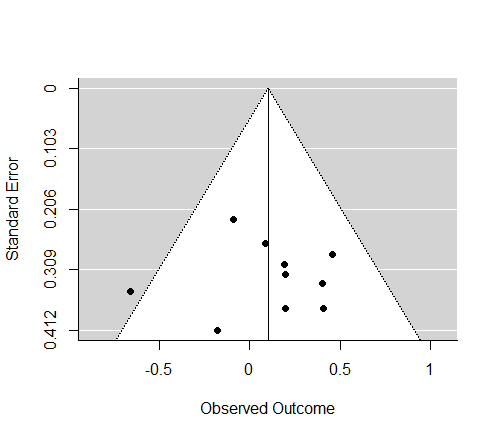

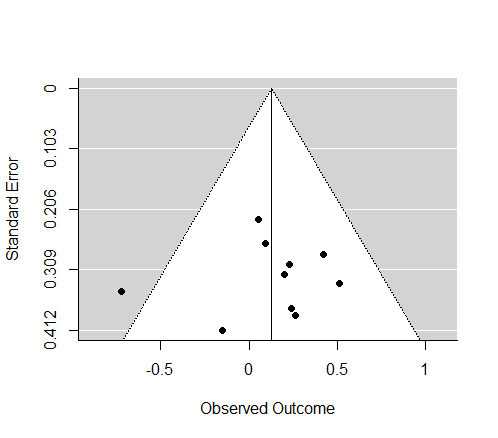

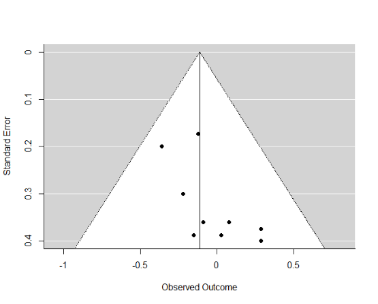

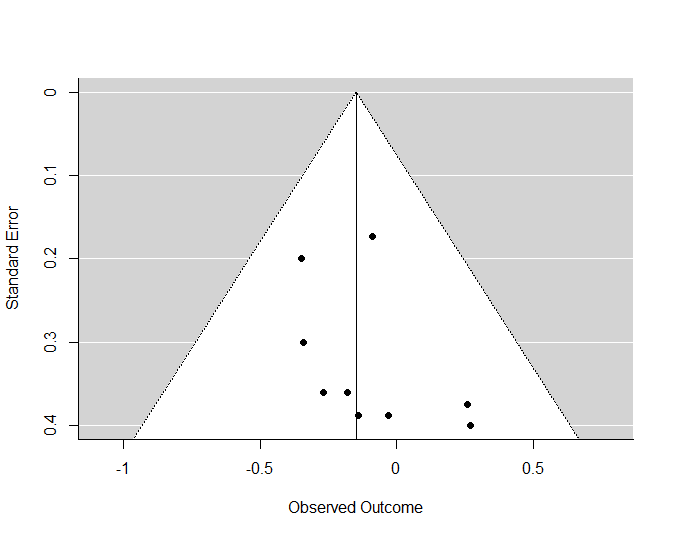

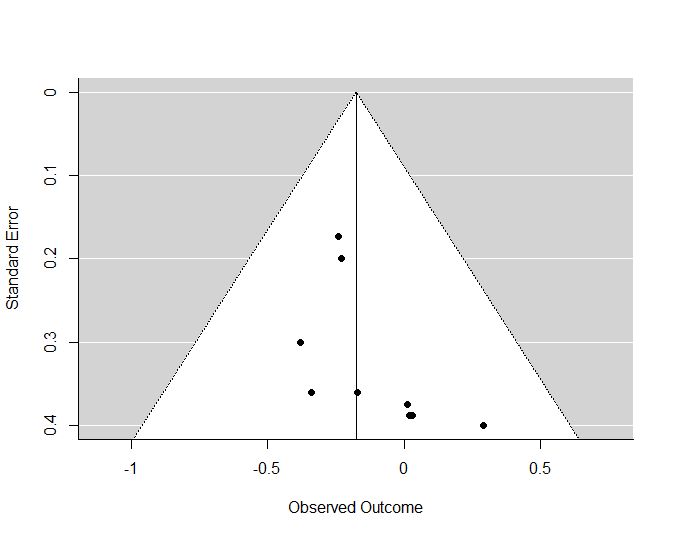

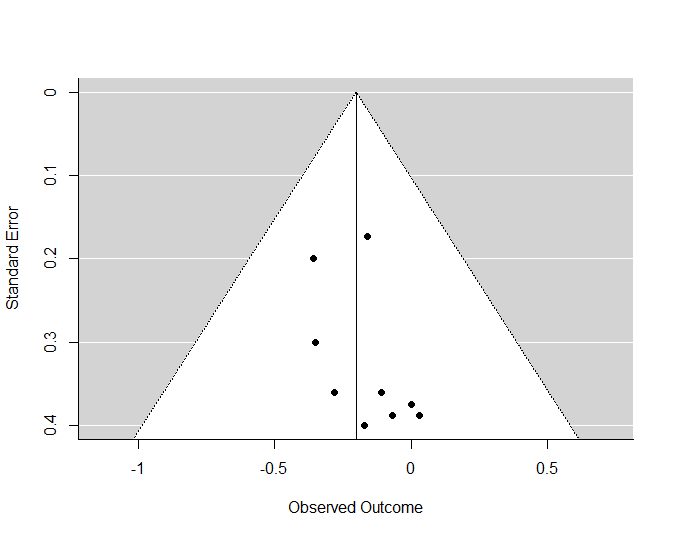

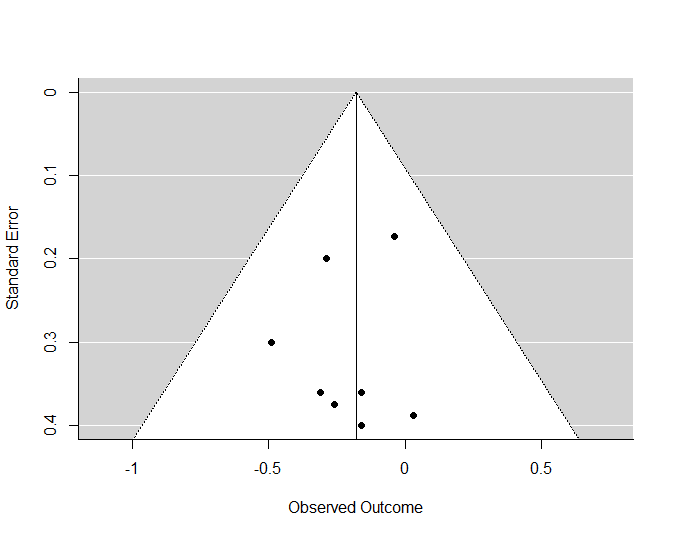


**Fig. S10** Funnel plots of effect sizes (0.75, 0.8, 0.85, 0.9, 0.95 quantiles) for each point-count study *Overall* and foraging guilds (*Insectivore*, *Frugivore* and *Omnivore*).

**OVERALL**

**INSECTIVORE**

**OMNIVORE**

**FRUGIVORE**

**0.75**

**0.95**

**0.90**

**0.85**

**0.80**


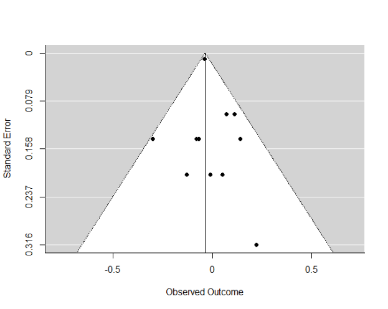

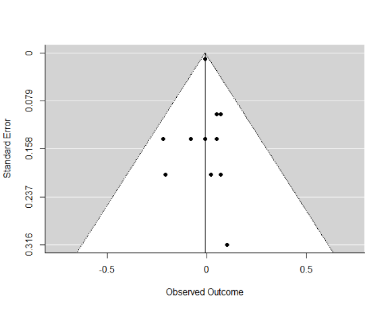

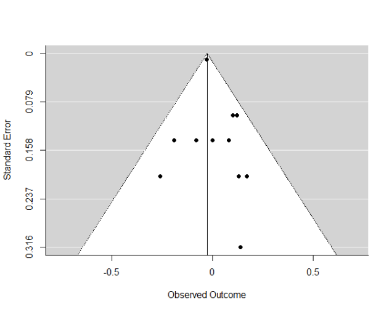

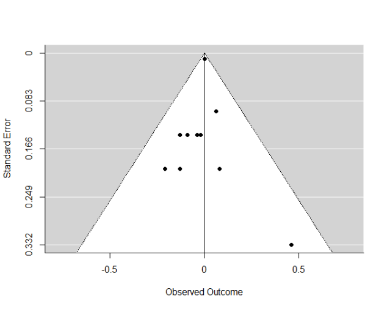

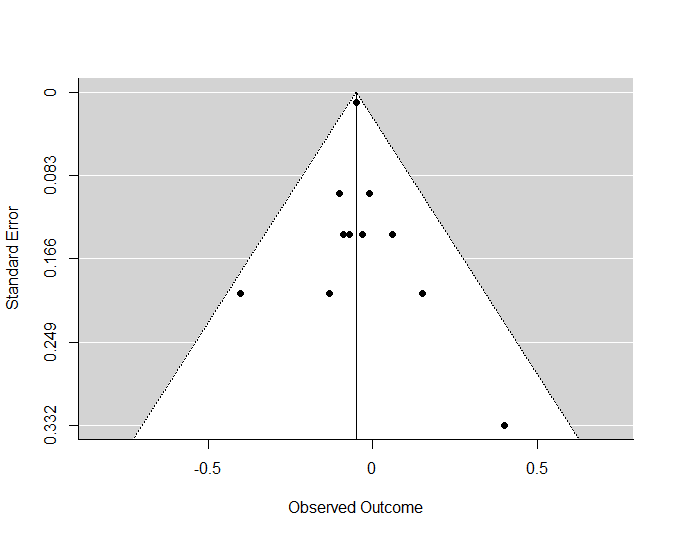

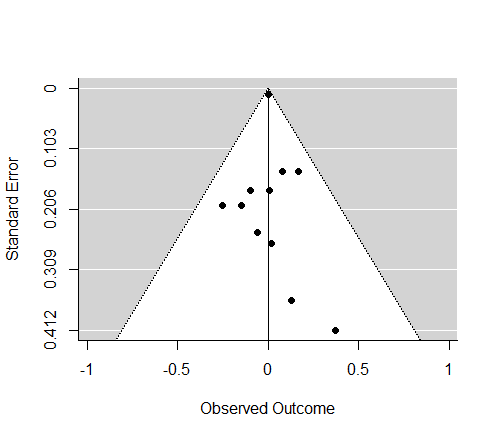

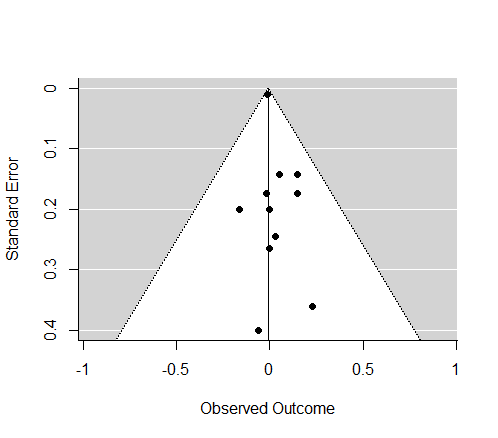

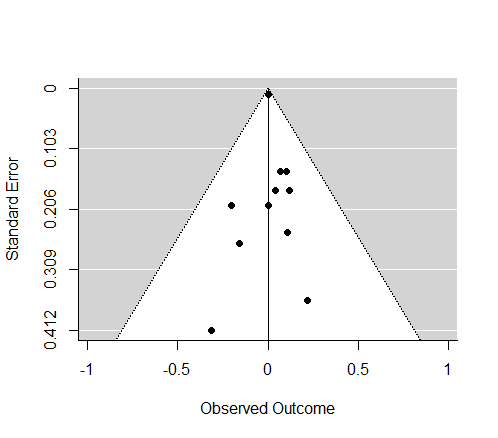

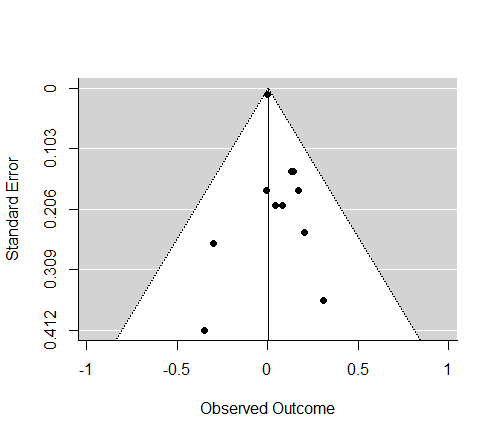

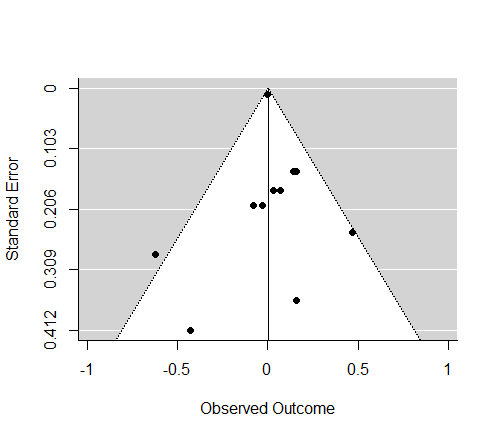

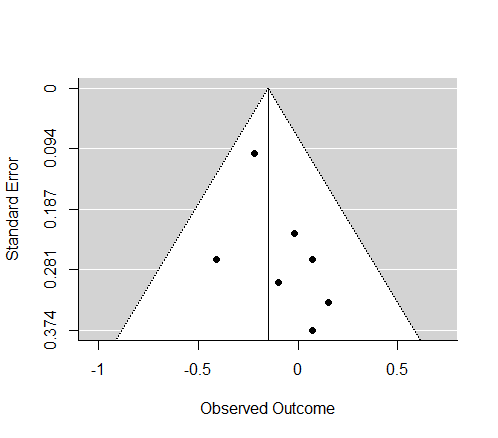

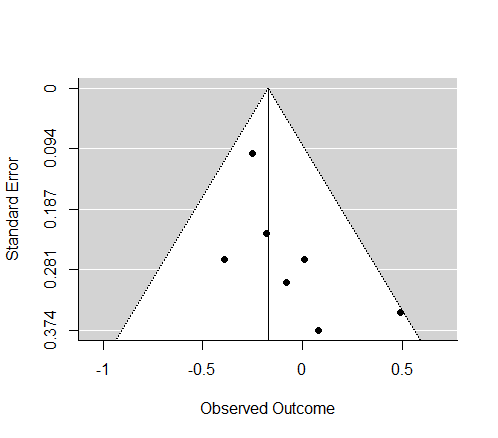

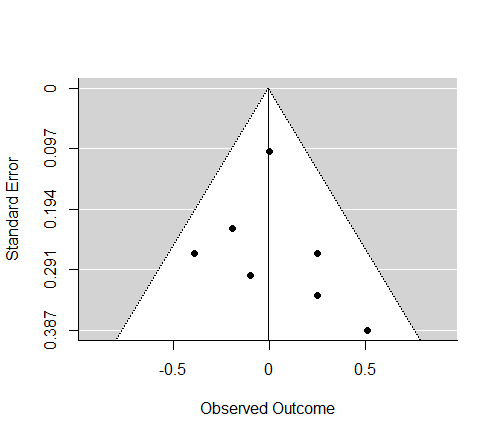

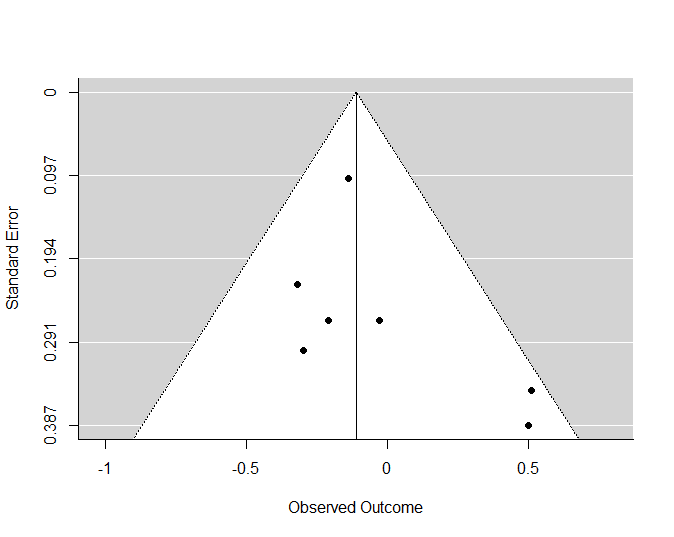

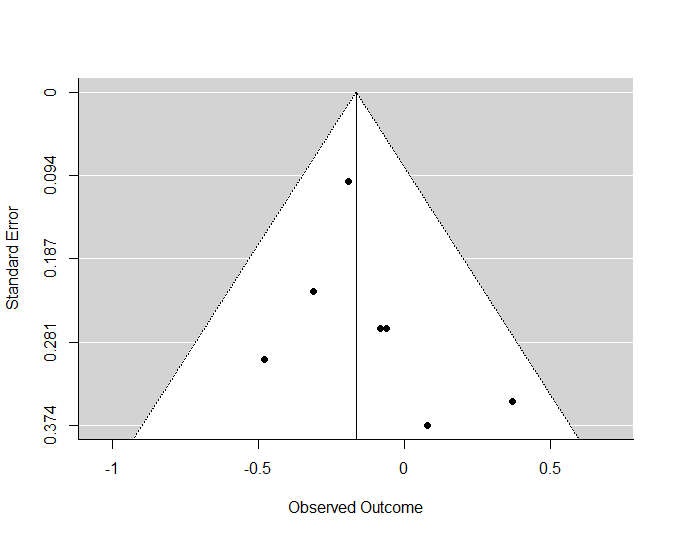

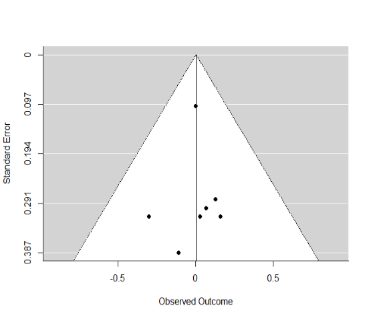

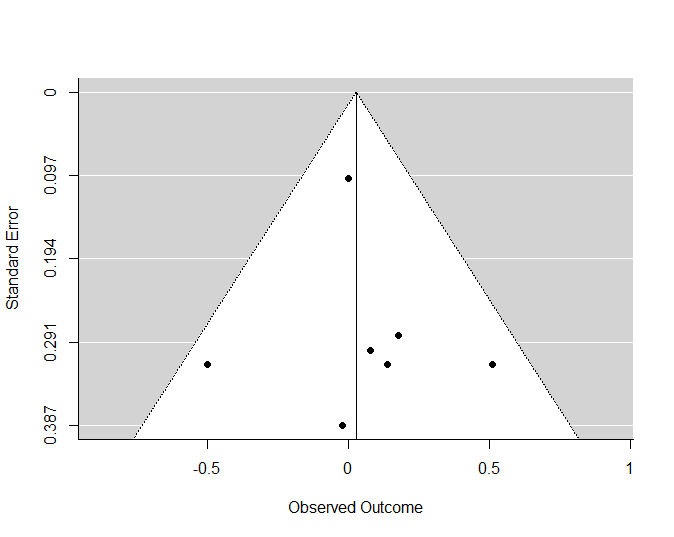

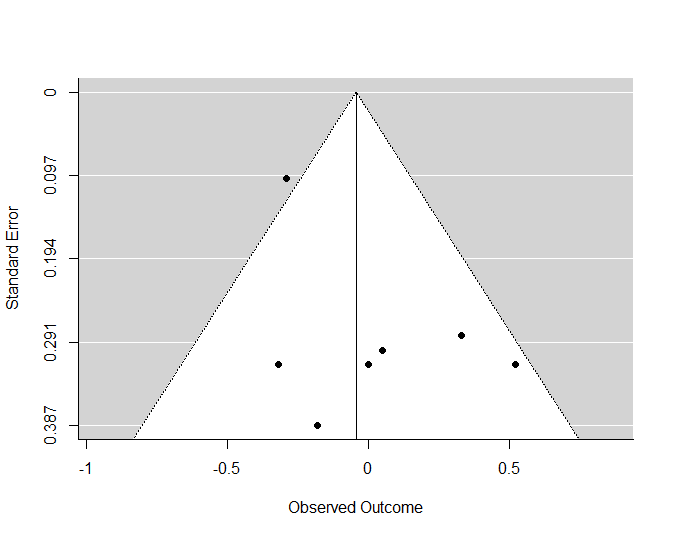

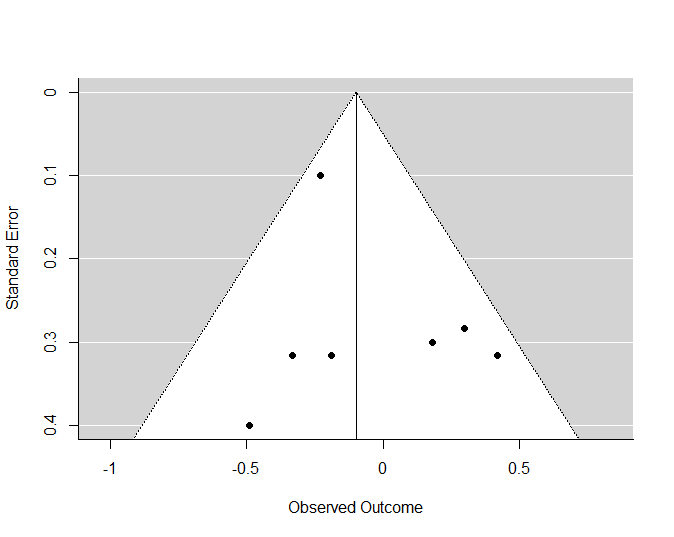

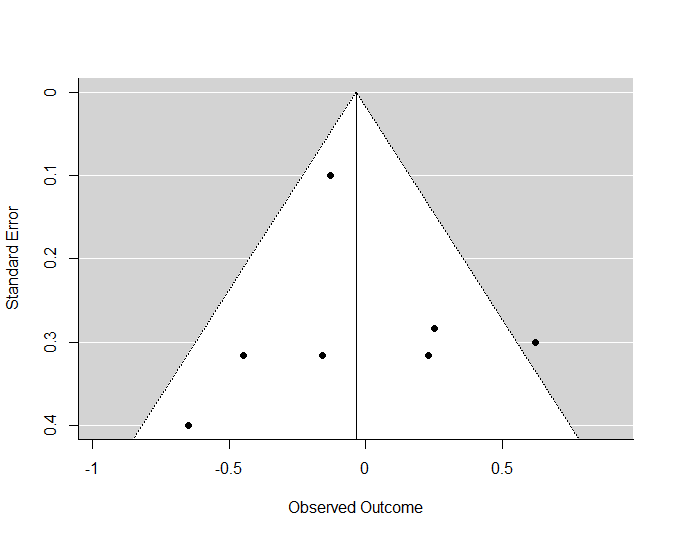

Supplement: Supplementary file 1 [file ECE3-10-2803-s001.docx]
